# Supplementary material for: Phospho-regulation of ATOH1 Is Required for Plasticity of Secretory Progenitors and Tissue Regeneration
Source: Cell Stem Cell. 2018 Sep 6;23(3):436–443.e7. doi: 10.1016/j.stem.2018.07.002 (PMC6138952; doi:10.1016/j.stem.2018.07.002)
Supplement: Document S1. Figures S1–S4 [file mmc1.pdf]

Cell Stem Cell, Volume *23*

Supplemental Information

Phospho-regulation of ATOH1 Is Required for Plasticity of Secretory Progenitors and Tissue Regeneration

Goran Tomic, Edward Morrissey, Sarah Kozar, Shani Ben-Moshe, Alice Hoyle, Roberta Azzarelli, Richard Kemp, Chandra Sekhar Reddy Chilamakuri, Shalev Itzkovitz, Anna Philpott, and Douglas J. Winton

Figure S1. Related to Figure 1.

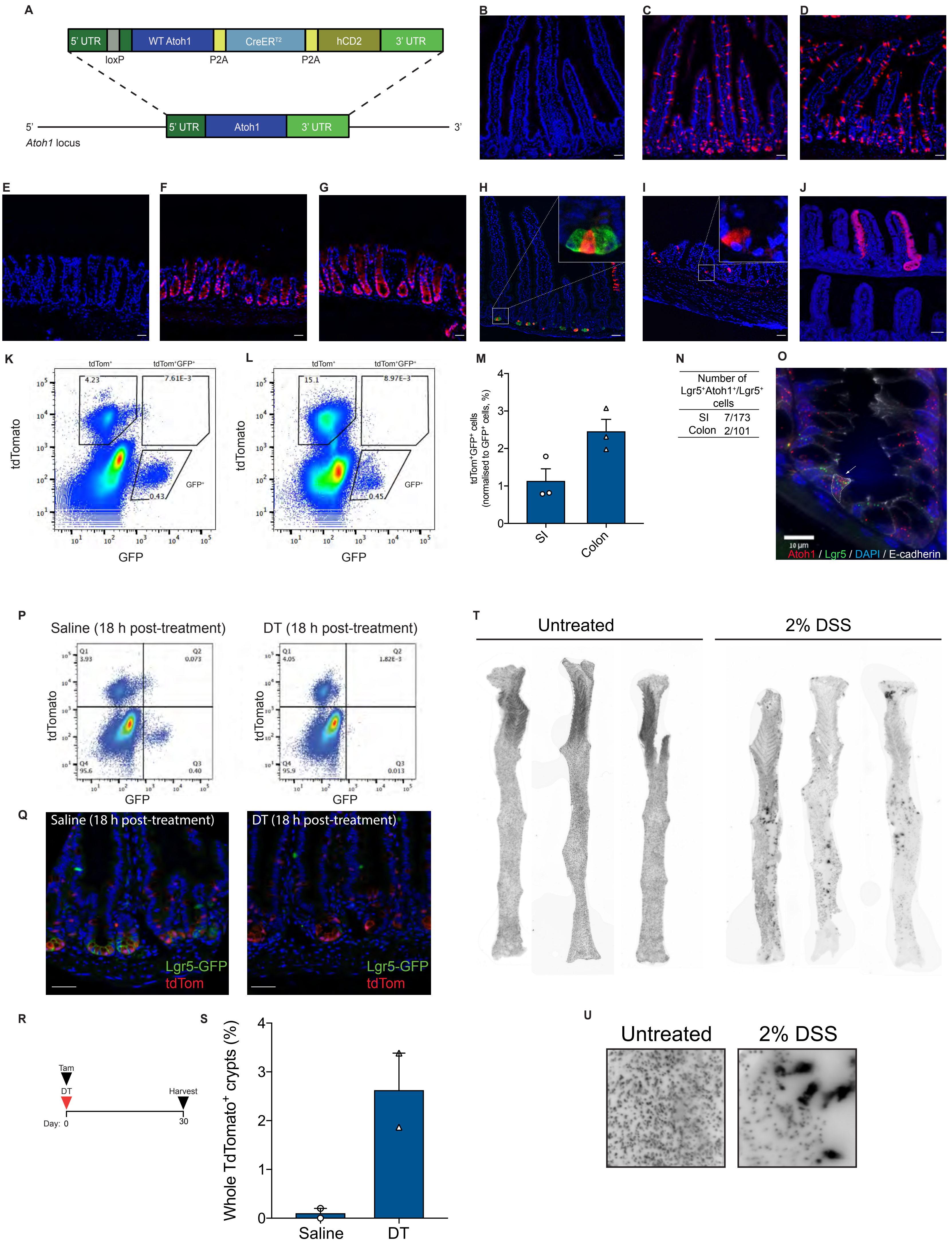

Figure S1. Generation of *Atoh1$^{(WT)CreERT2}$* mouse model and validation of lineage tracing in *R26R^{tdTom}* reporter line in homeostasis and injury, Related to Figure 1

(A) Schematic of the lineage tracing construct inserted into *Atoh1* genomic locus. (B, E) SI (B) and colon (E) of the uninduced animals. (C, F) Reporter positive cells in the SI (C) and colon (F), 24 h post-tamoxifen. (D, G) tdTom^{+} cells in the SI (D) and colon (G), 4 days post-tamoxifen. (H) Long-lived Lyz^{+} Paneth cells observed 30 days post-induction. (I) Long-lived reporter positive cells in the upper colon detected 30 days post-tamoxifen. (J) Labelled clonal event in the SI, 4 months post-tamoxifen. (K, L) Representative FACS graphs of tdTom^{+}GFP^{+} cells in SI (K), and colon (L), 24 hours post-tamoxifen. (M) Quantification of tdTom^{+}GFP^{+} cells in SI and colon. (N, O). Quantification (N) and a representative image (O) of Atoh1/Lgr5 transcript-positive cells in *WT* SI and colon (n=3). (P, Q) Ablation of Lgr5-GFP^{+} cells following administration of DT analysed by FACS (P) and microscopy (Q). (R) Schematic of the induction/injury protocol. (S) Quantification of tdTom^{+} clones in the SI without or with DT treatment (n=2 for each group). (T, U) Images of scanned colons showing tdTom^{+} crypts and multicrypt patches (MCPs) used for scoring in Figure 1U. Scale bars, 50 µm (B-J, Q); 10 µm (O). DT, diphtheria toxin.

Figure S2. Related to Figure 2.

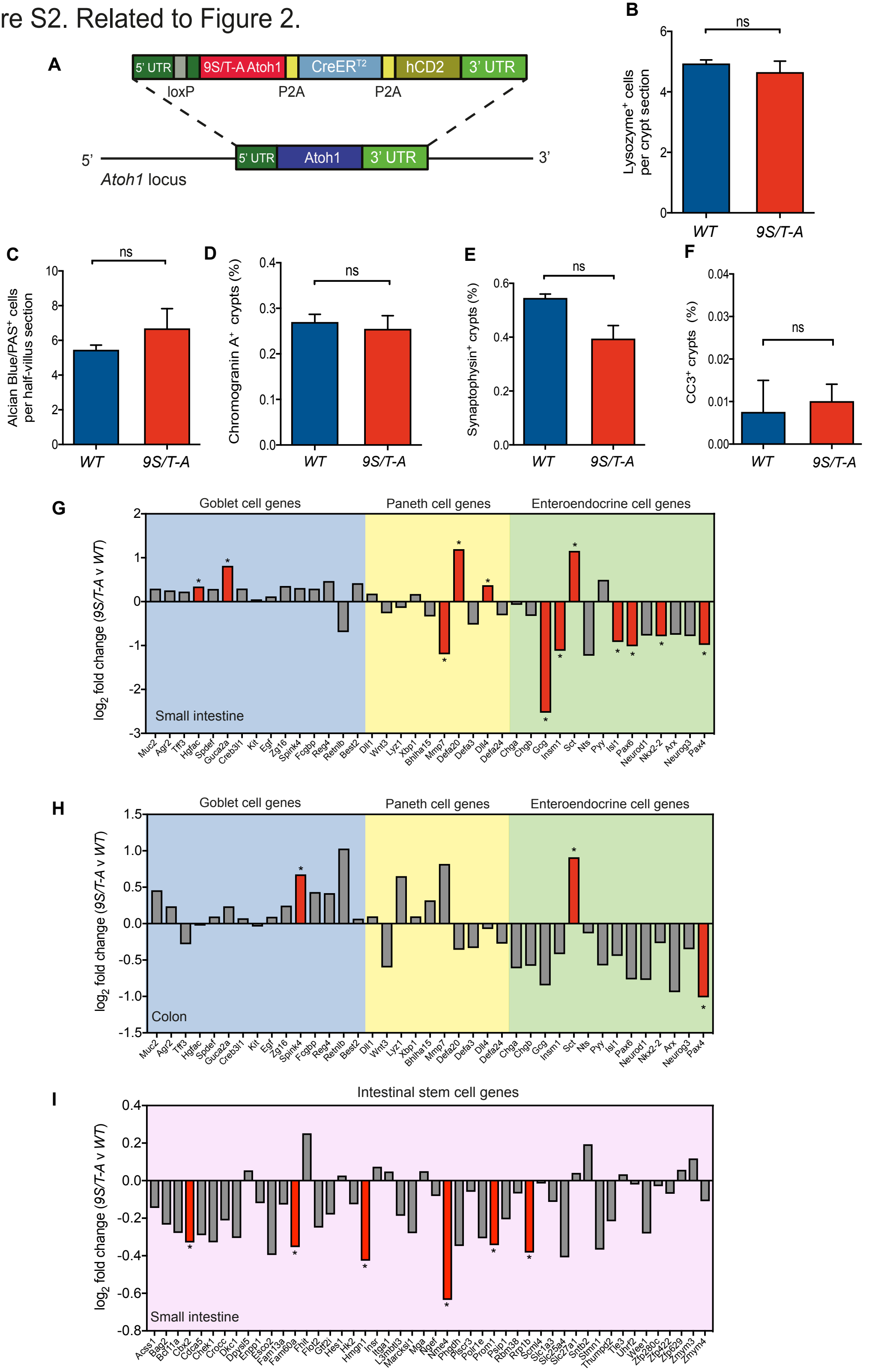

Figure S2. *Atoh1$^{(9S/T\text{-}A)CreERT2}$* mice do not exhibit abnormal intestinal secretory cell numbers, but show a change in gene expression profiles, Related to Figure 2

(A) Schematic representation of the *Atoh1$^{(9S/T\text{-}A)CreERT2}$* inserted into the *Atoh1* locus. (B-F) Quantification of differentiated small intestinal secretory lineage cells and apoptotic cells shows no difference between the genotypes. Paneth cells (B), goblet cells (C), enteroendocrine cells (D, E), and apoptotic cells (F) were scored (n=4 mice per group). (G-I) Expression of secretory genes selected based on known lineage-restricted pattern (Lo et al., 2017) in SI (G) and colon (H) of 9S/T-A Atoh1-expressing cells. (I) Expression of intestinal stem cell genes (Muñoz et al., 2012) in SI of 9S/T-A Atoh1-expressing cells. Genes that are significantly differentially expressed (FDR < 0.1) are shown in red, labelled with an asterisk. The results were generated from n=6 for both *WT* and *9S/T-A* groups.

Figure S3. Related to Figure 3.

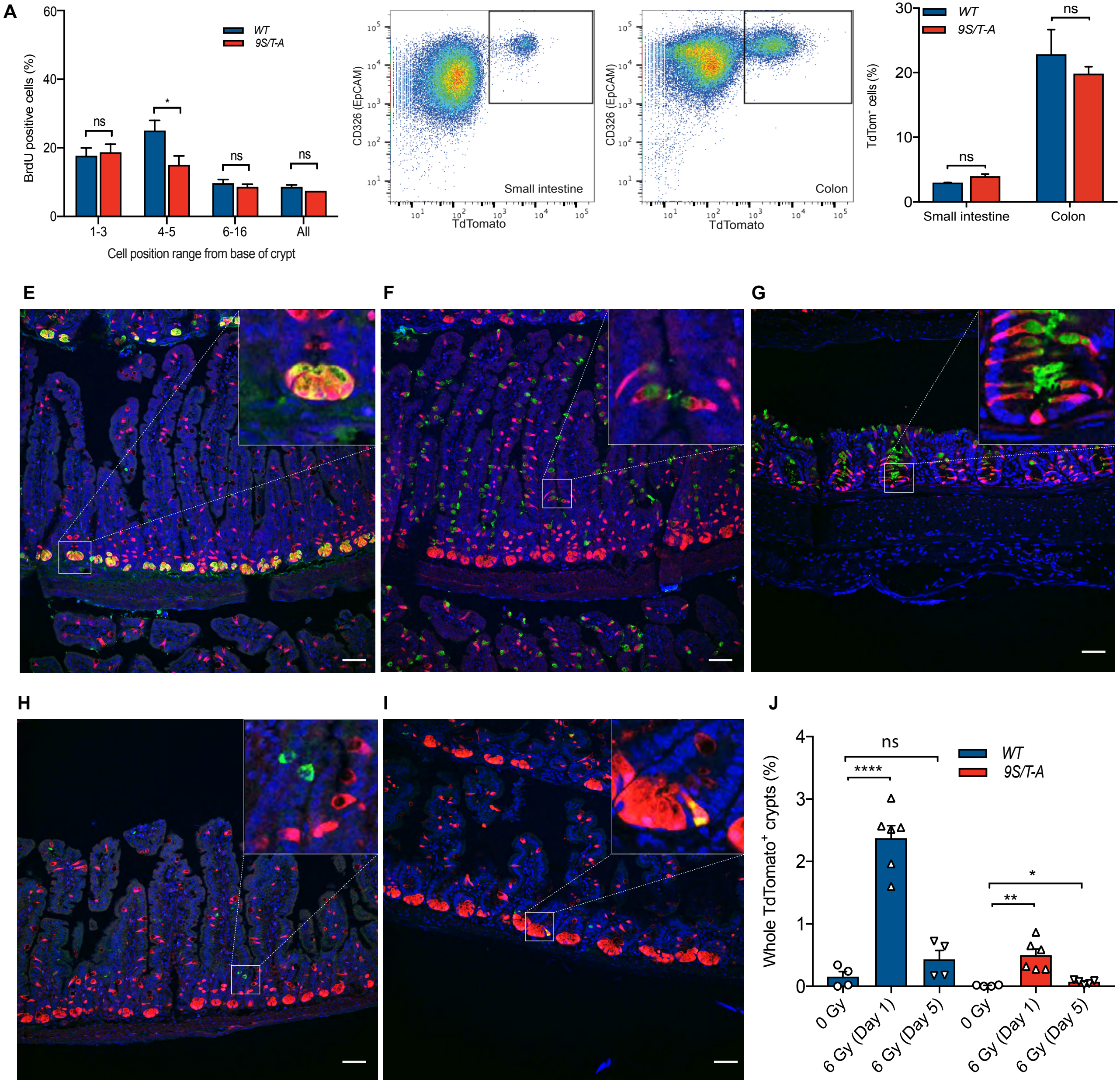

Figure S3. ***Atoh1***$^{(9S/T\text{-}A)CreERT2}$ **mice are phenotypically normal, but have a reduced clonogenic potential, Related to Figure 3.** (A) BrdU labelling index for a range of cell positions in colonic crypts (n=100 crypts, 4 mice per genotype; mean±s.e.m; * P=0.0101). (B, C) Representative flow cytometry plots of reporter positive cells in the SI (B) and colon (C). (D) Quantification of $tdTom^+$ cells in the SI and colon of the two mouse lines (n=3 per group, Mann Whitney test). (E-I) tdTom-labelling pattern in *9S/T-A* is identical to that of *WT*. Lyz^+ Paneth cells (E), $Muc2^+$ goblet cells in the SI (F), and colon (G) are positive for the reporter 24 h post-tamoxifen. $ChgA^+$ enteroendocrine cells do not co-label with tdTom after 24 h (H), but acquire the reporter by day 4 post-induction (I). (J) Comparison of the response to irradiation in WT Atoh1 and 9S/T-A small intestine. Scale bars, 50 µm. The data for WT Atoh1 are repeated from Figure 1O (Welch's T-test, **** P<0.001, ** P=0.0045, * P= 0.0122).

Figure S4. Related to Figure 4.

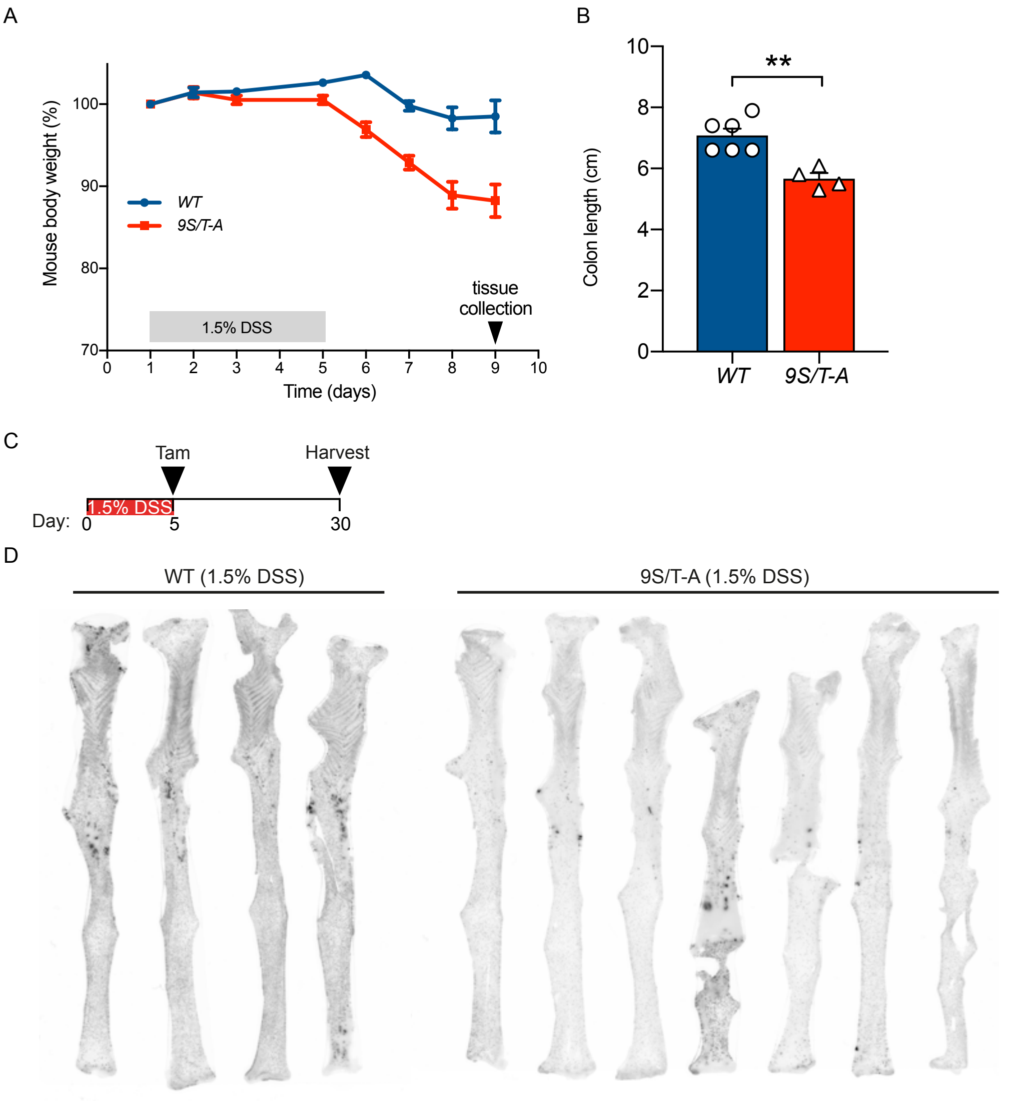

Figure S4. Characterisation of chemical colitis in *Atoh1*$^{(9S/T-A)CreERT2}$ mice, Related to Figure 4

(A) Mouse body weight difference between *WT* and *9S/T-A* mice on day 9 (n=6 (*WT*), n=4 (*9S/T-A*); related to Figure 4B and 4C). (B) Colon length following DSS treatment (n=6 (*WT*), n=4 (*9S/T-A*), mean±s.e.m, ** P=0.0048). (C) Lineage tracing protocol following DSS colitis. (D) Images of scanned colons used for quantification in Figures 4G and 4H.
