# Supplementary material for: Phospho-regulation of ATOH1 Is Required for Plasticity of Secretory Progenitors and Tissue Regeneration
Source: Cell Stem Cell. 2018 Sep 6;23(3):436–443.e7. doi: 10.1016/j.stem.2018.07.002 (PMC6138952; doi:10.1016/j.stem.2018.07.002)
Supplement: Document S2. Article plus Supplemental Information [file mmc5.pdf]

Cell Stem Cell

Short Article

# Phospho-regulation of ATOH1 Is Required for Plasticity of Secretory Progenitors and Tissue Regeneration

## Graphical Abstract

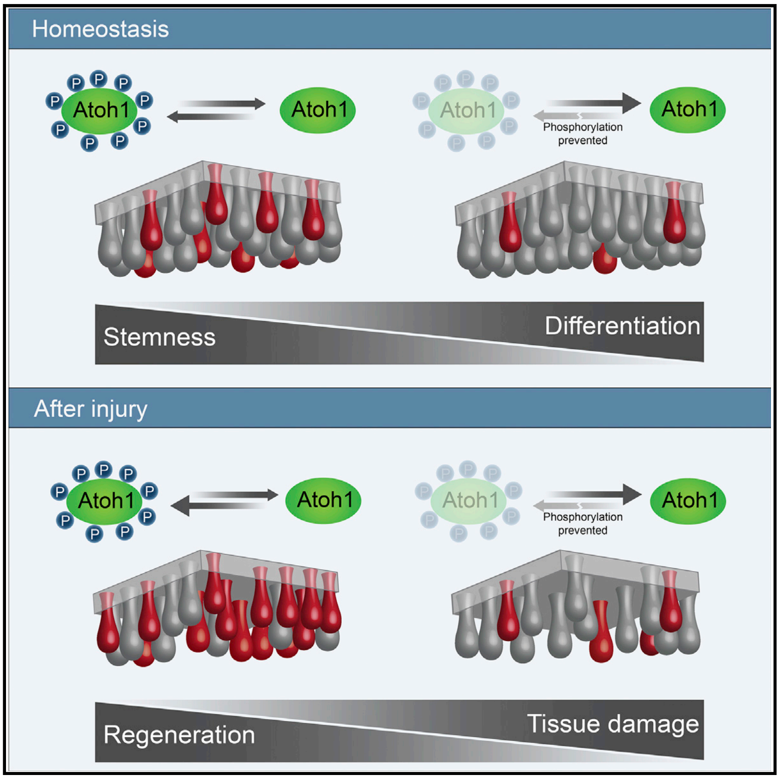


## Authors

Goran Tomic, Edward Morrissey, Sarah Kozar, ..., Shalev Itzkovitz, Anna Philpott, Douglas J. Winton

## Correspondence

ap113@cam.ac.uk (A.P.),
doug.winton@cruk.cam.ac.uk (D.J.W.)

## In Brief

Tomic et al. report that multisite phosphorylation of ATOH1 regulates the contribution of secretory progenitors to stem cell self-renewal in the small intestine and colon. With damage, the enhanced role of Atoh1$^+$ progenitors in mediating tissue repair is ablated in mice expressing phosphomutant ATOH1 and overall tissue regeneration is impaired.

## Highlights

- Atoh1$^+$ progenitors contribute to the stem cell pool in homeostasis and regeneration
- Multisite phosphorylation of ATOH1 regulates the plasticity of secretory progenitors
- Loss of phosphorylation of ATOH1 reduces clonogenic capacity of Atoh1$^+$ cells
- Phosphomutant ATOH1 mice are more susceptible to chemical colitis

Tomic et al., 2018, Cell Stem Cell *23*, 436–443
September 6, 2018 © 2018 The Authors. Published by Elsevier Inc.
https://doi.org/10.1016/j.stem.2018.07.002

CellPress

Cell Stem Cell
Short Article

# Phospho-regulation of ATOH1 Is Required for Plasticity of Secretory Progenitors and Tissue Regeneration

Goran Tomic,[1] Edward Morrissey,[2] Sarah Kozar,[1] Shani Ben-Moshe,[3] Alice Hoyle,[1] Roberta Azzarelli,[4,5] Richard Kemp,[1] Chandra Sekhar Reddy Chilamakuri,[1] Shalev Itzkovitz,[3] Anna Philpott,[4,5,*] and Douglas J. Winton[1,6,*]
[1]Cancer Research UK Cambridge Institute, University of Cambridge, Cambridge CB2 0RE, UK
[2]Weatherall Institute of Molecular Medicine, University of Oxford, John Radcliffe Hospital, Oxford OX3 9DS, UK
[3]Department of Molecular Cell Biology, Weizmann Institute of Science, Rehovot, Israel
[4]Department of Oncology, Hutchison/Medical Research Council (MRC) Research Centre, University of Cambridge, Cambridge CB2 0XZ, UK
[5]Wellcome-MRC Cambridge Stem Cell Institute, University of Cambridge, Cambridge CB2 1QR, UK
[6]Lead Contact
*Correspondence: ap113@cam.ac.uk (A.P.), doug.winton@cruk.cam.ac.uk (D.J.W.)
https://doi.org/10.1016/j.stem.2018.07.002

## SUMMARY

**The intestinal epithelium is largely maintained by self-renewing stem cells but with apparently committed progenitors also contributing, particularly following tissue damage. However, the mechanism of, and requirement for, progenitor plasticity in mediating pathological response remain unknown. Here we show that phosphorylation of the transcription factor Atoh1 is required for both the contribution of secretory progenitors to the stem cell pool and for a robust regenerative response. As confirmed by lineage tracing, Atoh1$^{+}$ cells (*Atoh1$^{(WT)CreERT2}$* mice) give rise to multilineage intestinal clones both in the steady state and after tissue damage. In a phosphomutant *Atoh1$^{(9S/T\text{-}A)CreERT2}$* line, preventing phosphorylation of ATOH1 protein acts to promote secretory differentiation and inhibit the contribution of progenitors to self-renewal. Following chemical colitis, Atoh1$^{+}$ cells of *Atoh1$^{(9S/T\text{-}A)CreERT2}$* mice have reduced clonogenicity that affects overall regeneration. Progenitor plasticity maintains robust self-renewal in the intestinal epithelium, and the balance between stem and progenitor fate is directly coordinated by ATOH1 multisite phosphorylation.**

## INTRODUCTION

Within the intestinal epithelium, cell generation occurs from phenotypically heterogenous stem cells residing at the base of glandular crypts (Vermeulen and Snippert, 2014). There is broad consensus that this heterogeneity reflects the combined behavior of active and reserve stem cells. The former dominates in homeostatic self-renewal and the latter following tissue damage. In homeostasis, rapidly cycling stem cells express the R-spondin receptor Lgr5. Reserve stem cell function is less defined and has been ascribed variously to a subset of quiescent Lgr5$^{+}$ cells (Barriga et al., 2017; Buczacki et al., 2013), progenitors committed to different intestinal lineages (van Es et al., 2012; Tetteh et al., 2016), and cells dependent on alternate pathways for stem cell maintenance (Takeda et al., 2011; Tian et al., 2011).

It has been demonstrated previously that cells of the secretory lineage possess reserve stem cell function in the small intestine (SI) epithelium in homeostasis and following tissue damage (van Es et al., 2012; Ishibashi et al., 2018; Yan et al., 2017; Yu et al., 2018). Subsequent to Delta-like expression (from Dll1 or Dll4), the basic helix-loop-helix (bHLH) transcription factor Atoh1 is upregulated, an event required for the creation of all secretory lineages within the epithelium (Yang et al., 2001). Atoh1$^{+}$ progenitors exhibit self-renewal and give rise to multilineage clones with higher frequency in homeostasis (Ishibashi et al., 2018) compared with previously described secretory Dll1$^{+}$ progenitors (van Es et al., 2012). This observation highlights a significant contribution of Atoh1$^{+}$ cells to the stem cell pool in the SI and colon. However, the mechanisms regulating intestinal plasticity and the nature of the relationship linking it to self-renewal remain unknown.

ATOH1 can be phosphorylated on multiple sites by cyclin-dependent kinases. Here we demonstrate that maintenance of the plasticity of committed secretory precursors allowing return to the stem compartment is dependent on the multisite phosphorylation of ATOH1, prevention of which inhibits Atoh1-mediated self-renewal and results in compromised regeneration following damage. We conclude that reversibility of the commitment to differentiate is dependent on post-translational control of ATOH1 and is required to maintain a robust stem cell population.

## RESULTS

### Atoh1$^{+}$ Cells Show Stem Cell Activity

Initially, to determine the extent to which Atoh1-expressing cells support stem cell maintenance in homeostasis, we generated a mouse (*Atoh1$^{(WT)CreERT2}$*) with an inducible *CreER$^{T2}$* downstream of the *Atoh1* coding sequence (Figure S1A). Acute lineage tracing demonstrated that tdTomato (tdTom) reporter

 © 2018 The Authors. Published by Elsevier Inc.
This is an open access article under the CC BY license (http://creativecommons.org/licenses/by/4.0/).

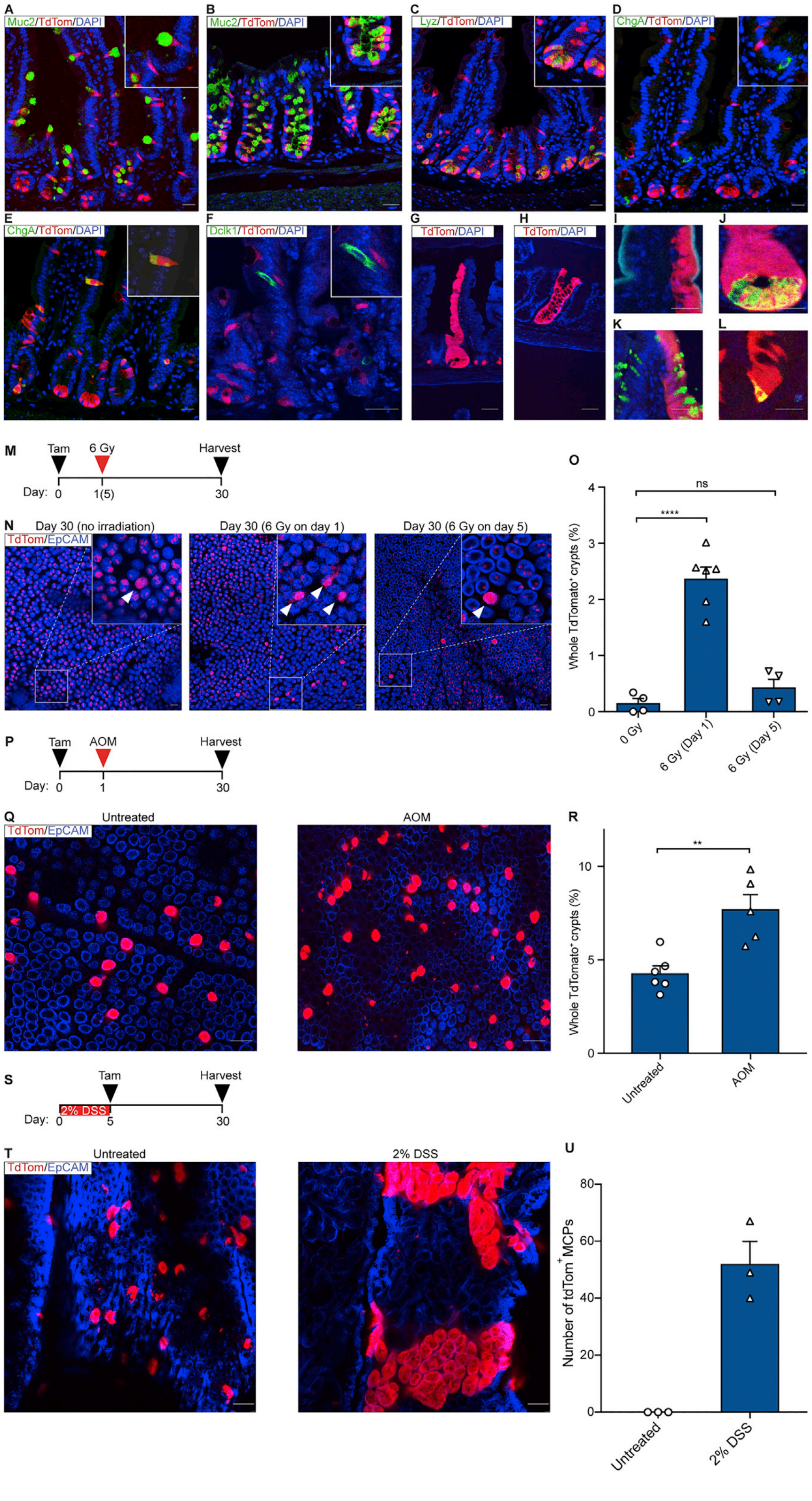


**Figure 1. Lineage Tracing of Atoh1$^{+}$ Cells in Homeostasis and after Injury**

(A–D) The tdTom reporter is detected in Muc2$^{+}$ goblet cells in the SI (A), colon (B), and Lyz$^{+}$ Paneth cells (C) but not in ChgA$^{+}$ enteroendocrine cells 24 hr post-tamoxifen (D). Muc2, Mucin 2; Lyz, Lysozyme; ChgA, Chromogranin A.

(E) ChgA$^{+}$ cells labeled with tdTom on day 4 after induction.

(F) Dclk1$^{+}$ tuft cells are not labeled with tdTom at 24 hr.

(G and H) Reporter-positive clone in the SI (G) and colon (H) 30 days following tamoxifen.

(I–L) tdTom$^{+}$ clones at 30 days are composed of alkaline phosphatase (Alpi$^{+}$) enterocytes (I), Paneth cells (J), goblet cells (K), and enteroendocrine cells (L).

(M, P, and S) Schematic of induction and injury protocol: irradiation (M), azoxymethane (AOM) (P), and dextran sodium sulfate (DSS) (S).

(N) Representative pictures of SI whole-mounts containing labeled crypts (arrowheads) 30 days post-induction.

(O) Quantification of tdTom$^{+}$ crypts in the SI (n = 4 for 0 Gy, n = 6 for 6 Gy [day 1], n = 4 for 6 Gy [day 5]).

(Q and T) Representative images of colonic crypts on day 30 post-tamoxifen and AOM (Q) or DSS treatment (T). Note the large tdTom$^{+}$ regenerative multicrypt patches (MCPs) associated with 2% DSS treatment (T).

(R) Quantification of reporter-positive crypts in the colon (n = 6 for untreated, n = 5 for AOM-treated).

(U) Quantification of tdTom$^{+}$ MCPs in untreated and DSS-treated colons (n = 3 for both groups).

Welch's t test was used in (O) (mean ± SEM, ****$p < 0.0001$) and Mann-Whitney test in (R) (mean ± SEM, **$p = 0.0087$). Scale bars, 50 μm (A–L) and 100 μm (N, Q, and T). See also Figure S1.

expression 24 hr following a single pulse of tamoxifen was restricted to secretory cells within the SI and colonic epithelium (Figures 1A–1D; Figures S1B–S1G). Mature Paneth and goblet cells were positive for the reporter whereas enteroendocrine cells (EECs) were not; the latter observation confirms that Atoh1 expression is not maintained in mature enteroendocrine cells (Bjerknes et al., 2012; Sommer and Mostoslavsky, 2014). However, by 4 days post-tamoxifen, enteroendocrine cells were also labeled (Figure 1E), indicating an origin from a secretory precursor that expressed Atoh1. Tuft cells were also not labeled with tdTom (Figure 1F). Individual Paneth cells remained labeled 4 weeks post-induction, reflecting their longevity (Figure S1H). Similar results were found in the colon, and long-lived secretory cells were also identified (Figure S1I). By 30 days post-induction, cohesive patches of reporter-positive cells that occupied all or a significant portion of entire crypts were present (Figures 1G and 1H) and continued to be observed after several months (Figure S1J). Immunostaining established the presence of goblet, Paneth, enteroendocrine, and absorptive

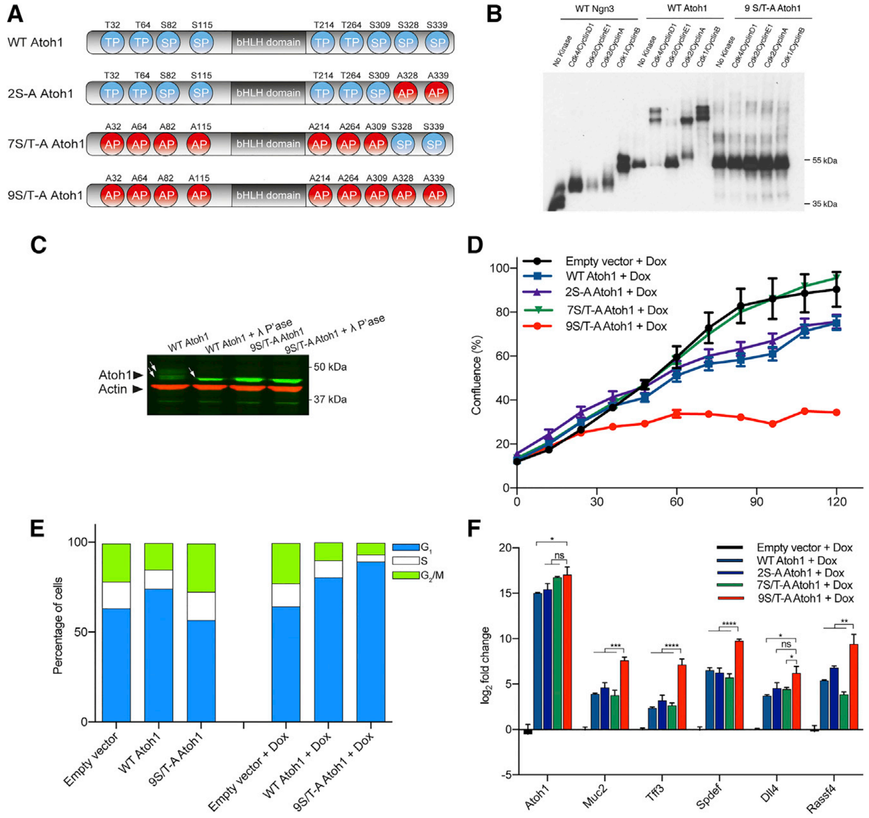


**Figure 2. Identification of a Hyperactive Phosphomutant ATOH1**

(A) Diagram depicting the location of proline-directed kinase motifs (serine-proline [SP] or threonine-proline [TP]) in Atoh1 protein and mutations of these sites into alanine in ATOH1 phosphomutants.

(B) *In vitro*-translated Atoh1 protein band-shift following incubation with different cyclin-dependent kinases (CDKs). Ngn3 was used as a positive control.

(C) WT ATOH1 bands (arrows) collapse following λ phosphatase treatment, demonstrating phosphorylation.

(D) DLD-1 cell proliferation following doxycycline (Dox)-induced expression of WT or phosphomutant Atoh1 (n = 3 biological replicates, 2 technical replicates, mean ± SEM).

(E) Cell cycle profile of uninduced and Dox-treated cells showing increased G1 and decreased S/M populations upon induction of *9S/T-A Atoh1*.

(F) Gene expression of Atoh1 and its target and secretory differentiation genes 72 hr after Dox induction of DLD-1 cells (n = 3 biological replicates, 2 technical replicates; Gapdh-normalized, mean ± SEM). Two-way ANOVA was used for statistical analysis; $^{*}p < 0.05$, $^{**}p < 0.01$, $^{***}p < 0.001$, $^{****}p < 0.0001$.

cells within reporter-positive epithelium, confirming their multilineage composition (Figures 1I–1L). These patterns are identical to those arising from individual marked intestinal stem cells (Vermeulen et al., 2013) and demonstrate a clonal origin from Atoh1$^{+}$ precursors. *Atoh1$^{(WT)CreERT2}$; Rosa26$^{TdTom}$* mice were then crossed onto *Lgr5$^{Gfp}$* reporter mice to investigate co-expression of Atoh1 and the intestinal stem cell marker Lgr5. The expression of Atoh1 and the tdTom reporter was identified in 1%–2% of Lgr5$^{+}$ (GFP$^{+}$) cells (Figures S1K–S1O), representing a likely intermediate state in the commitment process and candidate clonogenic population. Together, these results confirm that Atoh1 is appropriately expressed in mature Paneth and goblet cells but not enteroendocrine cells and that a proportion of Atoh1$^{+}$ progenitors are acting as long-term multipotential stem cells (Bjerknes et al., 2012; Sommer and Mostoslavsky, 2014; Ishibashi et al., 2018).

## Atoh1$^{+}$ Cells Contribute Directly to Epithelial Regeneration

The extent of reversibility of Atoh1$^{+}$ cell commitment was studied in the context of irradiation-induced tissue damage. Irradiation given 1 day after tamoxifen generated an increased number of tdTom$^{+}$ crypts at 30 days in the SI compared with unirradiated controls (16-fold increase, 2.37% versus 0.15%). This effect was abrogated when irradiation was given 5 days after tamoxifen (Figures 1M–1O), suggesting that regenerative potential is a property of progenitors arising *de novo* from the stem cell compartment and not of more mature secretory cells. Similarly, after targeted deletion of the bulk of Lgr5$^{+}$ stem cells using a diphtheria toxin approach (Figures S1P and S1Q), there was a 30-fold increase in the number of clones observed (Figures S1R and S1S). Adapting the assay to perform a similar analysis for the colonic epithelium and to circumvent that tissue's known radio-resistance (Cai et al., 1997), mice were treated with the colon-specific carcinogen azoxymethane (AOM) 1 day after tamoxifen treatment. Again, an increase in the frequency of tdTom$^{+}$ crypts was observed (Figures 1P–1R). Following dextran sodium sulfate (DSS)-induced colitis, multicrypt tdTom$^{+}$ patches (MCPs) were detected at the margins of regions of damage (Figures 1S–1U; Figures S1T and S1U). Together, these results suggest that Atoh1$^{+}$ cells directly contribute to regeneration following damage.

## Creating a Pro-secretory Phosphomutant ATOH1

Previous studies have indicated that multisite phosphorylation of bHLH proteins restrains cell cycle exit and limits differentiation, whereas, conversely, un(der)phosphorylation promotes these processes in the developing nervous system and pancreas (Ali et al., 2011, 2014; Azzarelli et al., 2017). However, a role for multisite phospho-regulation of bHLH proteins in adult homeostasis or tissue repair has not been reported. Hence, we hypothesized a potential role for ATOH1 phosphorylation in controlling the transition between stem and progenitor compartments both in homeostasis and under conditions of heightened proliferation following tissue damage. Cyclin-dependent kinases phosphorylate on serine-proline (SP) or threonine-proline (TP) residues. ATOH1 has 9 S/T-P sites available for phosphorylation (Figures 2A–2C). ATOH1 can be phosphorylated on many sites; we observed at least 5 distinct phospho-forms of ATOH1 after phosphorylation by different Cyclin and Cdk combinations (Figure 2B). We expressed forms of ATOH1 where S/T-P sites were mutated

to alanine-proline (AP) in colorectal cancer cells to determine the effect of ATOH1 phosphorylation on cell proliferation and on expression of markers of differentiation. The phosphorylation of two SP sites has previously been shown to destabilize the ATOH1 protein in the context of neuronal precursors (Forget et al., 2014). Although mutation of these two phospho-sites had a modest effect on ATOH1 activity, mutation of all 9 S/T-P sites was more effective at promoting enhanced cell cycle exit (Figures 2D and 2E). Additionally, the expression of secretory genes (Figure 2F) was enhanced after mutation of all 9 potential phosphorylation sites compared with both wild-type ATOH1, 2S-A ATOH1, and 7S/T-A ATOH1. These observations are consistent with multisite phospho-regulation of ATOH1 playing a significant role in controlling the balance between proliferation and differentiation, as described for other bHLH family members (Ali et al., 2011, 2014; Azzarelli et al., 2017).

## 9S/T-A Phosphomutant ATOH1 Promotes Secretory Maturation *In Vivo*

To investigate how preventing phosphorylation of ATOH1 affects progenitor-mediated self-renewal in homeostasis and repair, we substituted 9S/T-A ATOH1 for the wild-type form in its endogenous locus, generating a knockin mouse identical in design to *Atoh1*$^{(WT)CreERT2}$ but with the hyperactive phosphomutant *Atoh1*$^{(9S/T-A)CreERT2}$ allele (Figure S2A). Homozygous *Atoh1*$^{(9S/T-A)CreERT2}$ and control *Atoh1*$^{(WT)CreERT2}$ mice were generated. Phenotype analysis identified no gross differences between the two lines. Mice developed normally, and the overall morphological appearance of the epithelium remained unchanged. More detailed analysis found no difference in the number or distribution of the different secretory lineages or in the frequency of apoptotic cells (Figures S2B–S2F).

To investigate whether the 9S/T-A mutations affect secretory maturation after lineage specification, transcriptional profiling of secretory cells in the two lines was performed. First the expression profile of Atoh1$^+$ cells from *Atoh1*$^{(WT)CreERT2}$ mice was determined by comparing tdTom$^+$ (secretory) and tdTom$^-$ (absorptive) cells to define the baseline pro-secretory signature for both the colon and SI (Table S1). Next, the transcription profiles of tdTom$^+$ cells from wild-type and mutant mice (Table S2) were compared against this Atoh1$^+$ baseline and a published secretory signature (Lo et al., 2016). These gene set enrichment analyses (GSEAs) demonstrated a major pro-secretory shift in *Atoh1*$^{(9S/T-A)CreERT2}$ mice in both tissues and a strongly reduced intestinal stem cell signature compared with those from controls (Figures 3A–3E).

The pro-secretory nature of 9S/T-A-expressing cells arose from an overall elevation in pro-secretory transcripts for goblet and Paneth cell lineages (SI only). Atoh1$^+$ cells isolated from the SI of *Atoh1*$^{(9S/T-A)CreERT2}$ mice had a reduction in a subset of transcripts associated with enteroendocrine cells, indicating that ATOH1 phosphorylation influences their maturation (Figure S2G).

## Epithelial Proliferation and Clonogenicity Are Inhibited in *Atoh1*$^{9S/T-A}$ Mice

We next investigated whether the enhanced pro-secretory signature induced by prevention of multisite phosphorylation of ATOH1 is accompanied by changes in proliferation. Comparing proliferation between the two lines demonstrated a slight overall decrease in the total proliferative index of the crypts of *Atoh1*$^{(9S/T-A)CreERT2}$ mice in both the SI and colon, but that did not reach significance in the latter. More detailed spatial analysis within the crypt epithelium demonstrated that this effect was largely accounted for by a decrease in the proportion of cells in S phase in the epithelium of *9S/T-A* mutants in cell positions above the very base of the crypt and a reduction in the frequency of proliferative goblet cells (Figures 3F–3H; Figure S3A). This supports the interpretation that the phosphorylation of ATOH1 in cells immediately arising from the stem cell population limits Atoh1-dependent cell cycle exit to allow maintenance of proliferation in progenitors. Reciprocally, preventing this phosphorylation limits the ability to return to a proliferative stem and progenitor compartment. We next tested this hypothesis using a lineage tracing approach.

Lineage tracing and fluorescence-activated cell sorting (FACS) analysis established that the acute pattern of reporter expression and absolute number of tdTom$^+$ cells were the same in *Atoh1*$^{(9S/T-A)CreERT2}$ and controls (Figures S3B–S3I). However, lineage tracing at 30 days identified fewer epithelial clones in both the SI and colon than in *Atoh1*$^{(WT)CreERT2}$ mice (Figures 3I–3M). The 9S/T-A Atoh1$^+$ cells were also impaired in their ability to form tdTom$^+$ clones after radiation (Figure S3J). Together, the observations demonstrate that preventing phosphorylation of ATOH1 impairs the return of Atoh1$^+$ cells to the stem cell compartment and confirm a role for ATOH1 phosphorylation in maintenance of progenitor plasticity.

Previously, we and others have described that only a subset of competing stem cells drive increases in clone sizes that lead to surviving clones populating entire crypts (Kozar et al., 2013; Ritsma et al., 2014). To determine the net contribution of Atoh1$^+$ cells to this population, mathematical modeling was used to infer the proportion of the clonogenic fraction that is initially marked in *Atoh1*$^{(WT)CreERT2}$ and *Atoh1*$^{(9S/T-A)CreERT2}$ mice. In both the SI and colon, the contribution of Atoh1$^+$ progenitors to the stem cell pool is reduced in *9S/T-A* animals (Figures 3N–3Q). Between 1% and 2% of SI crypts in *Atoh1*$^{(WT)CreERT2}$ mice contain a single clonogenic stem cell derived from an Atoh1$^+$ progenitor, and this is reduced 5-fold in *Atoh1*$^{(9S/T-A)CreERT2}$ mice (Figures 3N and 3O). In the colon, values are higher, with the observed 4% wholly populated crypts (WPCs) and 5% partly populated crypts (PPCs) identified in *Atoh1*$^{(WT)CreERT2}$ mice 30 days post-induction requiring that initially 44% of crypts (1 in 15 active stem cells) contained an Atoh1$^+$-derived stem cell. This is reduced to 11% in *9S/T-A* mutant mice (Figures 3P and 3Q). Notably, these rates reflect the contribution of a single cohort of transient progenitors arising from the stem cell pool that are produced over 1 or 2 days.

## Compromised Epithelial Regeneration in *Atoh1*$^{9S/T-A}$ Mice

Although phosphorylation of ATOH1 clearly regulates reversion of secretory progenitors to the stem cell compartment, the absence of any other apparent phenotype in *9S/T-A Atoh1* mice suggests a limited requirement for such plasticity in homeostasis. We next investigated the role of ATOH1 phosphorylation in mounting a robust regenerative response following tissue damage. In the DSS-induced chemical colitis model,

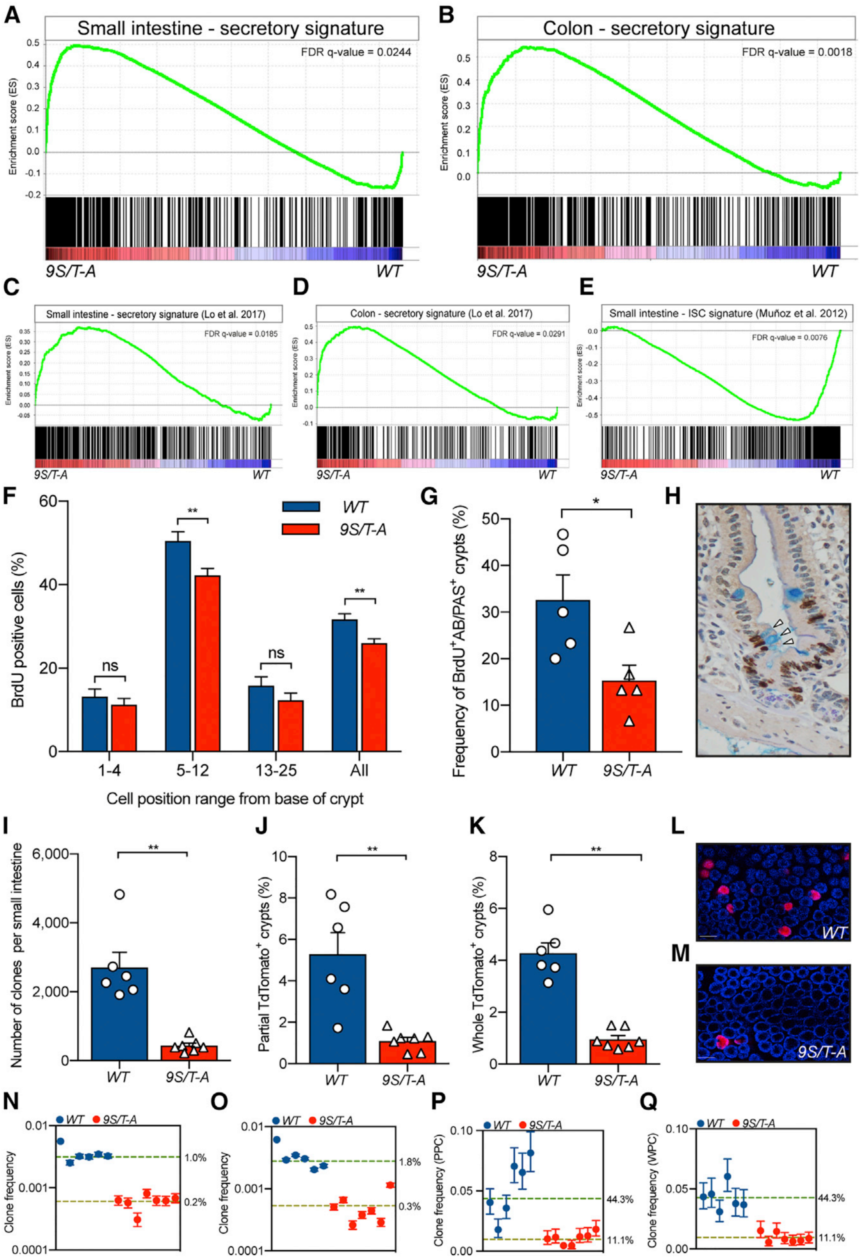


**Figure 3. 9S/T-A ATOH1 Promotes Secretory Maturation and Reduces Proliferation and the Number of Clonogenic Atoh1$^+$ Cells**

(A and B) Gene set enrichment analysis (GSEA) of the Atoh1$^+$ SI secretory signature (A) and colon (B) shows enrichment of secretory genes in 9S/T-A Atoh1 tdTom$^+$ cells.

(C and D) GSEA utilizing a published secretory transcriptome reveals an increase in the secretory gene signature in phosphomutant-expressing tdTom$^+$ cells in the SI (C) and colon (D).

(E) GSEA using a published intestinal stem cell (ISC) gene signature (Muñoz et al., 2012) shows a de-enrichment of ISC genes in the mutant SI progenitors.

(F and G) Bromodeoxyuridine (BrdU) labeling index for a range of cell positions in SI crypts (F) shows a reduction in proliferation above the crypt base (n = 100 crypts, 4 mice per genotype; mean ± SEM; **p = 0.0061 and 0.0015). Shown in (G) is the frequency of crypt-villus units containing at least one BrdU$^+$ goblet cell after a 24-hr BrdU pulse (n = 5 for both groups, *p = 0.0317).

(H) Representative image of a crypt-villus unit with a BrdU$^+$ Alcian blue (AB) and periodic acid-Schiff (PAS)$^+$ cell.

(I) Quantification of tdTom$^+$ clonal events in the SI (n = 6 [WT], n = 7 [*9S/T-A*], mean ± SEM, **p = 0.0012).

(J) Partly populated tdTom$^+$ crypts (PPCs) in the colon (n = 6 [WT], n = 7 [*9S/T-A*], mean ± SEM, **p = 0.0023).

(K) Wholly populated tdTom$^+$ crypts (WPCs) in WT and *9S/T-A* colons (n = 6 [WT], n = 7 [*9S/T-A*], mean ± SEM, **p = 0.0012; the same WT data are shown in Figure 1R because the experiment was done in parallel). All samples were collected 30 days after tamoxifen. Mann-Whitney test was used for all comparisons.

(L and M) Representative images of WT (L) and *9S/T-A* (M) colons scored in (J) and (K).

(N–Q) Inference of the proportion of the clonogenic fraction of labeled Atoh1$^+$ cells in the proximal SI (N), distal SI (O), colon PPCs (P), and colon WPCs (Q). The numbers next to the dotted lines indicate the inferred proportion of crypts that had one labeled stem cell. Scale bars, 75 μm.

*9S/T-A* mutant mice showed a greater sensitivity after treatment, with increased weight loss and slowed recovery (Figures 4A, S4A, and S4B). Analysis of this phenotype at the start of the regenerative phase (9 days after the start of DSS treatment) showed areas of ulceration that were more extensive in mice carrying the *9S/T-A* mutant (Figures 4B and 4C). At both 5 and 9 days, the proportion of secretory cells was identical for the two lines, and cell death was restricted to a few cells on the luminal surface, suggesting that the greater sensitivity does not arise from enhanced damage or deletion of secretory cells in *9S/T-A* mutant mice (Figures 4D–4F). However, lineage tracing 30 days following DSS treatment identified a reduced number and size of tdTom$^+$ regenerative patches in *9S/T-A* colons compared with the wild-type (WT) (Figures 4G, 4H, S4C, and S4D). Together, these results demonstrate that mice lacking the ability to phospho-regulate ATOH1 have compromised regenerative capacity following damage and that the contribution of Atoh1$^+$ progenitors is required for robust tissue repair.

## DISCUSSION

It is now accepted that cells with the capacity for self-renewal arise from a larger population whose members all have the same self-renewal potential subject to occupying available niches (Farin et al., 2016; Ritsma et al., 2014). Here we show that Atoh1$^+$ cells make a more substantial contribution to stem cell maintenance from cells committing to secretory differentiation than has been recognized so far (van Es et al., 2012;

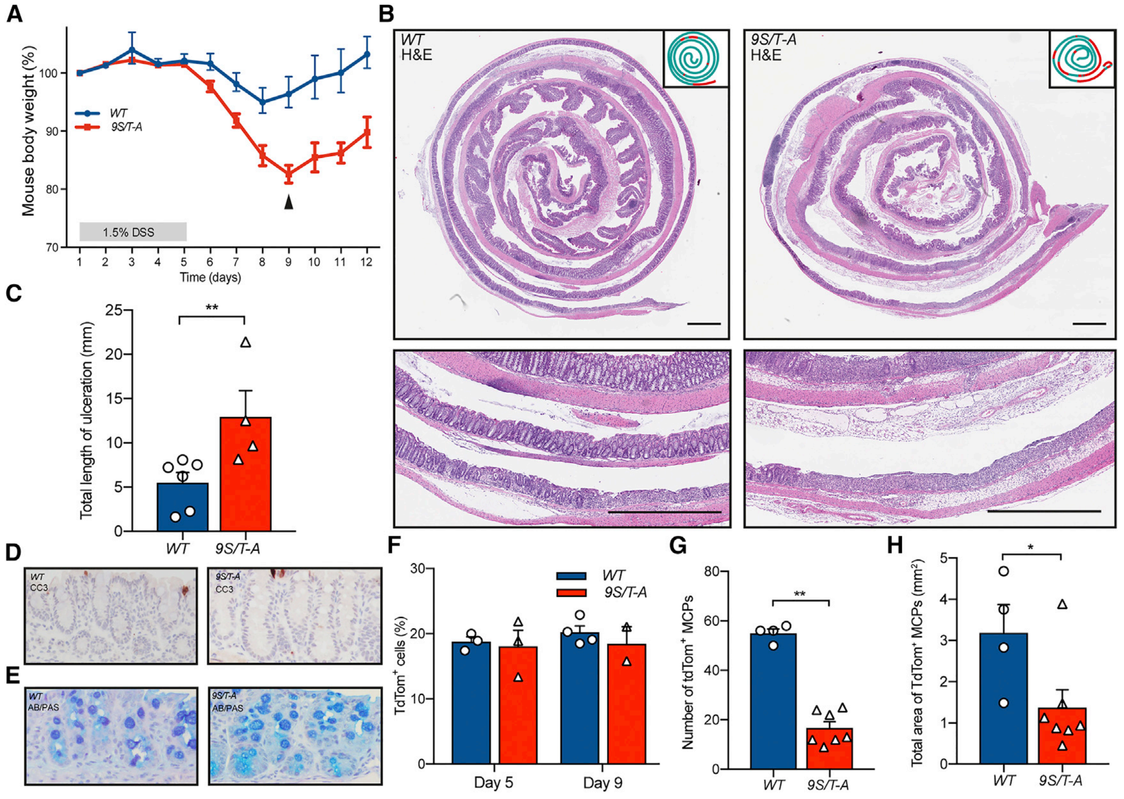


**Figure 4. *Atoh1*$^{(9S/T\text{-}A)CreERT2}$ Mice Are Sensitive to Chemical Colitis**

(A) Change in mouse body weight during and after DSS treatment (n = 5 [WT], n = 6 [*9S/T-A*]; two *9S/T-A* mice were euthanized on day 9 for health reasons, and one WT animal was taken for comparison [arrowhead]).

(B) Representative pictures and schematics of the colon on day 9, showing extensive loss of crypts in *9S/T-A* but not in the WT. Scale bars, 1 mm.

(C) Total length of colon ulceration on day 9 (n = 6 [WT], n = 4 [*9S/T-A*], mean ± SEM, **p = 0.0095).

(D and E) Representative images of the distal colon on day 5 of DSS treatment, showing apoptosis (D) and AB and PAS staining (E) in WT and *9S/T-A* animals.

(F) FACS analysis of the number of tdTom$^{+}$ cells during DSS-induced colitis.

(G and H) Analysis of the number (G) and total area (H) of tdTom$^{+}$ MCPs following 1.5% DSS (n = 4 [WT], n = 7 [*9S/T-A*], mean ± SEM, **p = 0.0061, *p = 0.0424). See also Figure S4.

Ishibashi et al., 2018). Self-renewal is therefore not solely a feature driven from a fixed pool of stem cells but, rather, involves dynamic interchange between progenitors and stem cells in the steady state.

Transcription factors of the bHLH family have been extensively studied as master regulators of cell fate commitment and differentiation in a wide variety of tissues, including the nervous system and intestine (Ali et al., 2011, 2014; Yang et al., 2001). However, in recent years, additional roles for these proteins are emerging in direct co-ordination of cell cycle and differentiation events, particularly during embryonic development (Castro and Guillemot, 2011). Intestinal homeostasis in many ways represents an ongoing development-like hierarchical process where crypts are maintained by stem cells feeding a proliferating progenitor compartment that gives rise to a variety of mature cell types. What is now also emerging is a picture of significant plasticity where cells expressing Atoh1, previously thought to represent a population that has undergone secretory commitment, can nevertheless revert to “stemness” and repopulate the entire crypt with surprisingly high frequency. The mechanisms controlling this plasticity have been unclear. Here we determine that the balance between stem and progenitor fate behavior in the intestine can be controlled by Atoh1 multisite phosphorylation under normal homeostatic conditions.

Control of proliferation and differentiation by modulation of bHLH protein phosphorylation is emerging as an important mechanism in development of the nervous system and the pancreas (Cleaver, 2017; Guillemot and Hassan, 2017). We now demonstrate that multisite phosphorylation is also required to restrain irreversible commitment of secretory precursors in the adult homeostatic gut and so to maintain their ability to repopulate the stem cell compartment. Consistent with this, a phosphomutant form of ATOH1 enhances the expression of gene sets associated with a more mature secretory phenotype in colorectal carcinoma cells. Interestingly, in the homeostatic gut, despite Atoh1$^{+}$ cells normally supplying up to 1 in 15 cells in the stem cell compartment, the phosphomutant Atoh1-expressing intestine is essentially phenotypically normal, indicating that plasticity from the secretory to the stem compartment is not essential in normal homeostasis. However, intestinal regeneration after damage is substantially compromised by an inability to phosphorylate ATOH1.

Taken together, our results indicate that multisite phosphorylation of ATOH1 is used to dynamically regulate the return of secretory precursors to the stem cell compartment, which facilitates the capacity of the epithelium as a whole to respond rapidly to changes in the cellular environment. Damaging the intestine using irradiation or DSS (van Es et al., 2012; Ishibashi et al., 2018) leads to acute cell damage and death, followed by proliferative regeneration that produces new cells for tissue repair. Activation of cyclin-dependent kinases (CDKs) and mitogen-activated protein kinases (MAPKs) in rapidly proliferating cells undergoing regeneration would result in enhanced phosphorylation of ATOH1, restraining further progression down the secretory lineage and supporting re-entry of Atoh1-expressing cells into a stem-like state. The post-translational regulation of ATOH1 by proline-directed kinases to modulate the balance between proliferation and differentiation in response to changing tissue demands in the adult intestinal epithelium echoes the regulation and effect of other bHLH proteins as development progresses (Hardwick et al., 2015).

The secretory fate choice mediated by ATOH1, a master regulator, is not irreversible differentiation; rather, it is entry into a plastic state through which progression is regulated by post-translational modifications. Functionally, the implications are likely to be that post-translational modifications facilitate rapid cellular responses by allowing reversal of commitment or varying its extent or rate. Progenitor plasticity is not merely an incidental acquired behavior following damage but plays an integral part in tissue restoration and requires post-translational regulation of ATOH1.

## STAR★METHODS

Detailed methods are provided in the online version of this paper and include the following:


- KEY RESOURCES TABLE
- CONTACT FOR REAGENT AND RESOURCE SHARING
- EXPERIMENTAL MODEL AND SUBJECT DETAILS
  - Mice
  - Cell Lines
- METHOD DETAILS
  - Cloning of mouse knock-in constructs
  - ES cell targeting
  - Mouse genotyping
  - Creation of doxycycline inducible DLD-1 cells
  - Treatment of animals
  - Crypt fractionation and single cell preparation
  - Flow cytometry
  - Whole-mount preparation
  - Whole-mount scanning and quantification
  - Antibody staining
  - Quantification of crypts in whole-mounts
  - Immunostainings
  - Single molecule FISH
  - Analysis of gut sections
  - Colon ulceration scoring
  - RNA isolation
  - Gene expression analysis
  - RNA sequencing
  - Western blotting
  - *In vitro* kinase assay
  - Cell proliferation and cell cycle analysis
  - RNA sequencing analysis
  - Secretory signature gene list
  - Gene Set Enrichment Analysis (GSEA)
- QUANTIFICATION AND STATISTICAL ANALYSIS
  - Computational analysis
  - Model fitting
  - Statistical analysis
- DATA AND SOFTWARE AVAILABILITY


## SUPPLEMENTAL INFORMATION

Supplemental Information includes four figures and three tables and can be found with this article online at https://doi.org/10.1016/j.stem.2018.07.002.


## ACKNOWLEDGMENTS

This work was supported by Cancer Research UK (to G.T., E.M., A.H., R.K., and D.J.W.) and Wellcome Trust grant 103805 (to G.T. and D.J.W.). A.P. and R.A. were funded by MRC research grant MR/K018329/1 and grant MR/L021129/1, the Rosetrees Trust, and the Stoneygate Trust and received core support from the Wellcome-MRC Cambridge Stem Cell Institute. We thank S. Taylor for DLD-1 Flp-In T-Rex cells and D. Perera for plasmids. We thank the Histopathology core and Biological Resources Unit at the CRUK Cambridge Institute for technical support and the members of the Winton and Philpott labs for their help.


## AUTHOR CONTRIBUTIONS

Conceptualization, D.J.W., A.P., G.T., and R.K.; Methodology, G.T., D.J.W., and A.P.; Formal Analysis, G.T., E.M., and C.S.R.C.; Investigation, G.T., S.K., S.B.-M., A.H., and R.A.; Writing – Original Draft, D.J.W., A.P., and G.T.; Writing – Review & Editing, G.T., D.J.W., and A.P.; Visualization, G.T.; Supervision, D.J.W., A.P., and S.I.; Project Administration, D.J.W. and A.P.; Funding Acquisition, D.J.W. and A.P.

## DECLARATION OF INTERESTS

The authors declare no competing interests.


Received: February 2, 2018
Revised: May 25, 2018
Accepted: July 6, 2018
Published: August 9, 2018


## REFERENCES


Ali, F., Hindley, C., McDowell, G., Deibler, R., Jones, A., Kirschner, M., Guillemot, F., and Philpott, A. (2011). Cell cycle-regulated multi-site phosphorylation of Neurogenin 2 coordinates cell cycling with differentiation during neurogenesis. Development *138*, 4267–4277.

Ali, F.R., Cheng, K., Kirwan, P., Metcalfe, S., Livesey, F.J., Barker, R.A., and Philpott, A. (2014). The phosphorylation status of Ascl1 is a key determinant of neuronal differentiation and maturation in vivo and in vitro. Development *141*, 2216–2224.

Azzarelli, R., Hurley, C., Sznurkowska, M.K., Rulands, S., Hardwick, L., Gamper, I., Ali, F., McCracken, L., Hindley, C., McDuff, F., et al. (2017). Multi-site Neurogenin3 Phosphorylation Controls Pancreatic Endocrine Differentiation. Dev. Cell *41*, 274–286.e5.

Barriga, F.M., Montagni, E., Mana, M., Mendez-Lago, M., Hernando-Momblona, X., Sevillano, M., Guillaumet-Adkins, A., Rodriguez-Esteban, G., Buczacki, S.J.A., Gut, M., et al. (2017). Mex3a Marks a Slowly Dividing Subpopulation of Lgr5+ Intestinal Stem Cells. Cell Stem Cell *20*, 801–816.e7.

Bjerknes, M., Khandanpour, C., Möröy, T., Fujiyama, T., Hoshino, M., Klisch, T.J., Ding, Q., Gan, L., Wang, J., Martín, M.G., and Cheng, H. (2012). Origin of the brush cell lineage in the mouse intestinal epithelium. Dev. Biol. *362*, 194–218.

Buczacki, S.J.A., Zecchini, H.I., Nicholson, A.M., Russell, R., Vermeulen, L., Kemp, R., and Winton, D.J. (2013). Intestinal label-retaining cells are secretory precursors expressing Lgr5. Nature *495*, 65–69.

Cai, W.B., Roberts, S.A., Bowley, E., Hendry, J.H., and Potten, C.S. (1997). Differential survival of murine small and large intestinal crypts following ionizing radiation. Int. J. Radiat. Biol. *71*, 145–155.

Carpenter, B., Gelman, A., Hoffman, M.D., Lee, D., Goodrich, B., Betancourt, M., Brubaker, M., Guo, J., Li, P., and Riddell, A. (2017). Stan : A Probabilistic Programming Language. J. Stat. Softw. *76*, 1–32.

Castro, D.S., and Guillemot, F. (2011). Old and new functions of proneural factors revealed by the genome-wide characterization of their transcriptional targets. Cell Cycle *10*, 4026–4031.

Cleaver, O. (2017). β Cell Renewal versus Differentiation: Slow and Steady Wins the Race. Dev. Cell *41*, 223–225.

Farin, H.F., Jordens, I., Mosa, M.H., Basak, O., Korving, J., Tauriello, D.V.F., de Punder, K., Angers, S., Peters, P.J., Maurice, M.M., and Clevers, H. (2016). Visualization of a short-range Wnt gradient in the intestinal stem-cell niche. Nature *530*, 340–343.

Forget, A., Bihannic, L., Cigna, S.M., Lefevre, C., Remke, M., Barnat, M., Dodier, S., Shirvani, H., Mercier, A., Mensah, A., et al. (2014). Shh signaling protects Atoh1 from degradation mediated by the E3 ubiquitin ligase Huwe1 in neural precursors. Dev. Cell *29*, 649–661.

Guillemot, F., and Hassan, B.A. (2017). Beyond proneural: emerging functions and regulations of proneural proteins. Curr. Opin. Neurobiol. *42*, 93–101.

Hardwick, L.J.A., Ali, F.R., Azzarelli, R., and Philpott, A. (2015). Cell cycle regulation of proliferation versus differentiation in the central nervous system. Cell Tissue Res. *359*, 187–200.

Ishibashi, F., Shimizu, H., Nakata, T., Fujii, S., Suzuki, K., Kawamoto, A., Anzai, S., Kuno, R., Nagata, S., Ito, G., et al. (2018). Contribution of ATOH1$^{+}$ Cells to the Homeostasis, Repair, and Tumorigenesis of the Colonic Epithelium. Stem Cell Reports *10*, 27–42.

Itzkovitz, S., Lyubimova, A., Blat, I.C., Maynard, M., van Es, J., Lees, J., Jacks, T., Clevers, H., and van Oudenaarden, A. (2011). Single-molecule transcript counting of stem-cell markers in the mouse intestine. Nat. Cell Biol. *14*, 106–114.

Kim, D., Pertea, G., Trapnell, C., Pimentel, H., Kelley, R., and Salzberg, S.L. (2013). TopHat2: accurate alignment of transcriptomes in the presence of insertions, deletions and gene fusions. Genome Biol. *14*, R36.

Kozar, S., Morrissey, E., Nicholson, A.M., van der Heijden, M., Zecchini, H.I., Kemp, R., Tavaré, S., Vermeulen, L., and Winton, D.J. (2013). Continuous clonal labeling reveals small numbers of functional stem cells in intestinal crypts and adenomas. Cell Stem Cell *13*, 626–633.

Lallemand, Y., Luria, V., Haffner-Krausz, R., and Lonai, P. (1998). Maternally expressed PGK-Cre transgene as a tool for early and uniform activation of the Cre site-specific recombinase. Transgenic Res. *7*, 105–112.

Lo, Y.-H., Chung, E., Li, Z., Wan, Y.-W., Mahe, M.M., Chen, M.-S., Noah, T.K., Bell, K.N., Yalamanchili, H.K., Klisch, T.J., et al. (2016). Transcriptional Regulation by ATOH1 and its Target SPDEF in the Intestine. Cell. Mol. Gastroenterol. Hepatol. *3*, 51–71.

Lopez-Garcia, C., Klein, A.M., Simons, B.D., and Winton, D.J. (2010). Intestinal stem cell replacement follows a pattern of neutral drift. Science *330*, 822–825.

Love, M.I., Huber, W., and Anders, S. (2014). Moderated estimation of fold change and dispersion for RNA-seq data with DESeq2. Genome Biol. *15*, 550.

Lyubimova, A., Itzkovitz, S., Junker, J.P., Fan, Z.P., Wu, X., and van Oudenaarden, A. (2013). Single-molecule mRNA detection and counting in mammalian tissue. Nat. Protoc. *8*, 1743–1758.

Muñoz, J., Stange, D.E., Schepers, A.G., van de Wetering, M., Koo, B.-K., Itzkovitz, S., Volckmann, R., Kung, K.S., Koster, J., Radulescu, S., et al. (2012). The Lgr5 intestinal stem cell signature: robust expression of proposed quiescent '+4′ cell markers. EMBO J. *31*, 3079–3091.

Ritsma, L., Ellenbroek, S.I.J., Zomer, A., Snippert, H.J., de Sauvage, F.J., Simons, B.D., Clevers, H., and van Rheenen, J. (2014). Intestinal crypt homeostasis revealed at single-stem-cell level by in vivo live imaging. Nature *507*, 362–365.

Snippert, H.J., van der Flier, L.G., Sato, T., van Es, J.H., van den Born, M., Kroon-Veenboer, C., Barker, N., Klein, A.M., van Rheenen, J., Simons, B.D., and Clevers, H. (2010). Intestinal crypt homeostasis results from neutral competition between symmetrically dividing Lgr5 stem cells. Cell *143*, 134–144.

Sommer, C.A., and Mostoslavsky, G. (2014). RNA-Seq analysis of enteroendocrine cells reveals a role for FABP5 in the control of GIP secretion. Mol. Endocrinol. *28*, 1855–1865.

Subramanian, A., Tamayo, P., Mootha, V.K., Mukherjee, S., Ebert, B.L., Gillette, M.A., Paulovich, A., Pomeroy, S.L., Golub, T.R., Lander, E.S., and Mesirov, J.P. (2005). Gene set enrichment analysis: a knowledge-based approach for interpreting genome-wide expression profiles. Proc. Natl. Acad. Sci. USA *102*, 15545–15550.

Takeda, N., Jain, R., LeBoeuf, M.R., Wang, Q., Lu, M.M., and Epstein, J.A. (2011). Interconversion between intestinal stem cell populations in distinct niches. Science *334*, 1420–1424.

Tetteh, P.W., Basak, O., Farin, H.F., Wiebrands, K., Kretzschmar, K., Begthel, H., van den Born, M., Korving, J., de Sauvage, F., van Es, J.H., et al. (2016). Replacement of Lost Lgr5-Positive Stem Cells through Plasticity of Their Enterocyte-Lineage Daughters. Cell Stem Cell *18*, 203–213.

Tian, H., Biehs, B., Warming, S., Leong, K.G., Rangell, L., Klein, O.D., and de Sauvage, F.J. (2011). A reserve stem cell population in small intestine renders Lgr5-positive cells dispensable. Nature *478*, 255–259.

van Es, J.H., Sato, T., van de Wetering, M., Lyubimova, A., Yee Nee, A.N., Gregorieff, A., Sasaki, N., Zeinstra, L., van den Born, M., Korving, J., et al. (2012). Dll1+ secretory progenitor cells revert to stem cells upon crypt damage. Nat. Cell Biol. *14*, 1099–1104.

Vermeulen, L., and Snippert, H.J. (2014). Stem cell dynamics in homeostasis and cancer of the intestine. Nat. Rev. Cancer *14*, 468–480.

Vermeulen, L., Morrissey, E., van der Heijden, M., Nicholson, A.M., Sottoriva, A., Buczacki, S., Kemp, R., Tavaré, S., and Winton, D.J. (2013). Defining stem cell dynamics in models of intestinal tumor initiation. Science *342*, 995–998.

Yan, K.S., Gevaert, O., Zheng, G.X.Y., Anchang, B., Probert, C.S., Larkin, K.A., Davies, P.S., Cheng, Z.F., Kaddis, J.S., Han, A., et al. (2017). Intestinal Enteroendocrine Lineage Cells Possess Homeostatic and Injury-Inducible Stem Cell Activity. Cell Stem Cell *21*, 78–90.e6.

Yang, Q., Bermingham, N.A., Finegold, M.J., and Zoghbi, H.Y. (2001). Requirement of Math1 for secretory cell lineage commitment in the mouse intestine. Science *294*, 2155–2158.

Yu, S., Tong, K., Zhao, Y., Balasubramanian, I., Yap, G.S., Ferraris, R.P., Bonder, E.M., Verzi, M.P., and Gao, N. (2018). Paneth Cell Multipotency Induced by Notch Activation following Injury. Cell Stem Cell *23*, 46–59.e5.

# STAR★METHODS

## KEY RESOURCES TABLE

| REAGENT or RESOURCE | SOURCE | IDENTIFIER |
|---|---|---|
| Antibodies | | |
| Mouse Monoclonal anti-Atoh1 | Developmental Studies Hybridoma Bank | Cat# Math1 (Atoh1): RRID:AB_10805299 |
| Rabbit Polyclonal anti-β-actin | Abcam | Cat# ab8227: RRID:AB_2305186 |
| IRDye 800CW Goat anti-Mouse IgG (H + L) | LI-COR Biosciences | Cat# P/N 925-32210: RRID:AB_2687825 |
| IRDye 680LT Goat anti-Rabbit IgG (H + L) | LI-COR Biosciences | Cat# P/N 925-68021: RRID:AB_2713919 |
| Rat Anti-Mouse CD326 (Ep-CAM) Monoclonal Antibody, Alexa Fluor 488 Conjugated, Clone G8.8 | BioLegend | Cat# 118210: RRID:AB_1134099 |
| Sheep Polyclonal BrdU Antibody | Abcam | Cat# ab1893: RRID:AB_302659 |
| Rabbit Polyclonal Anti Human Lysozyme | Dako | Cat# A0099: RRID:AB_2341230 |
| Biotin-SP-AffiniPure Donkey Anti-Sheep IgG (H+L) | Jackson ImmunoResearch Labs | Cat# 713-066-147: RRID:AB_2340717 |
| Biotin-SP-AffiniPure Donkey Anti-Rabbit IgG (H+L) | Jackson ImmunoResearch Labs | Cat# 711-065-152: RRID:AB_2340593 |
| Rabbit Anti-Chromogranin A Polyclonal Antibody | Abcam | Cat# ab15160: RRID:AB_301704 |
| Rabbit Polyclonal Anti-Synaptophysin Antibody | Millipore | Cat# AB9272: RRID:AB_570874 |
| Rabbit Polyclonal anti-DCAMKL1 Antibody | Abcam | Cat# ab31704: RRID:AB_873537 |
| Rabbit Anti-Human Lysozyme Polyclonal Antibody, FITC Conjugated | Dako | Cat# F037201: RRID:AB_578661 |
| Rabbit Anti-Mucin 2 Polyclonal Antibody | Santa Cruz Biotechnology | Cat# sc-15334: RRID:AB_2146667 |
| Donkey anti-Rabbit IgG (H+L) Secondary Antibody, Alexa Fluor 488 | Thermo Fisher Scientific | Cat# A-21206: RRID:AB_2535792 |
| Bacterial and Virus Strains | | |
| RP24-77K22 Bacterial Artificial Chromosome | BACPAC Resources Center | N/A |
| Chemicals, Peptides, and Recombinant Proteins | | |
| Lambda Protein Phosphatase (Lambda PP) | New England Biolabs | Cat# P0753S |
| Doxycycline Hydrochloride, Ready Made Solution | Sigma-Aldrich | Cat# D3072 |
| Tet Approved FBS | Clontech Laboratories | Cat# 631101 |
| Tamoxifen | Sigma-Aldrich | Cat# T5648 |
| Dextran Sulfate Sodium Salt | MP Biomedicals | Cat# 02160110 |
| Critical Commercial Assays | | |
| In-Fusion HD Cloning Kit | Clontech Laboratories | Cat# 639648 |
| TruSeq Stranded mRNA Library Prep Kit | Illumina | Cat# 20020595 |
| Deposited Data | | |
| RNA sequencing data | This paper | GEO: GSE115416 |
| Mendeley Data | This paper | https://doi.org/10.17632/vgvdv5b949.1 |
| Experimental Models: Cell Lines | | |
| DLD-1 Flp-In T-Rex cell line | Laboratory of Stephen Taylor | N/A |
| Experimental Models: Organisms/Strains | | |
| $Atoh1^{(WT)CreERT2}$ | This paper | N/A |
| $Atoh1^{(9S/T-A)CreERT2}$ | This paper | N/A |

(*Continued on next page*)

**Continued**

| REAGENT or RESOURCE | SOURCE | IDENTIFIER |
|---|---|---|
| Oligonucleotides | | |
| Left integration arm, Atoh1 locus, forward primer GGACAGGCGGGAACCACAGA | This paper | N/A |
| Left integration arm, Atoh1 locus, reverse primer TTGTCAACACGAGCTGGTCGAA | This paper | N/A |
| Right integration arm, Atoh1 locus, forward primer CAACACAACCCTGACCTGTG | This paper | N/A |
| Right integration arm, Atoh1 locus, reverse primer CCCTAACCAGTGTGCCCTTA | This paper | N/A |
| Left integration arm, DLD-1, forward primer AGTCAG CAACCATAGTCCCG | This paper | N/A |
| Left integration arm, DLD-1, reverse primer TTCTGCGGGCGATTTGTGTA | This paper | N/A |
| Right integration arm, DLD-1, forward primer TAAACGGCCACAAGTTCAGC | This paper | N/A |
| Left integration arm, DLD-1, reverse primer CGGGCCTCTTCGCTATTACG | This paper | N/A |
| Atoh1 genotyping forward primer TTTGTTGTTGTTGTTCGGGG | This paper | N/A |
| Atoh1 genotyping reverse primer TCTTTTACCTCAGCCCACTCTT | This paper | N/A |
| Software and Algorithms | | |
| TopHat2 | Kim et al., 2013 | N/A |
| DESeq2 | Love et al., 2014 | N/A |
| GSEA | Subramanian et al., 2005 | http://software.broadinstitute.org/gsea/index.jsp |

## CONTACT FOR REAGENT AND RESOURCE SHARING

Further information and requests for resources and reagents should be directed to and will be fulfilled by the Lead Contact, Douglas J. Winton (doug.winton@cruk.cam.ac.uk).

## EXPERIMENTAL MODEL AND SUBJECT DETAILS

### Mice

Mice used in this study were 8-16 weeks old males and females of C57BL/6 background. The mice were housed under controlled conditions (temperature (21 ± 2°C), humidity (55 ± 10%), 12 h light/dark cycle) in a specific-pathogen-free (SPF) facility (tested according to the recommendations for health monitoring by the Federation of European Laboratory Animal Science Associations). The animals had unrestricted access to food and water, were not involved in any previous procedures and were test naive. All experiments were carried out on homozygous $Atoh1^{(WT)CreERT2}$ and $Atoh1^{(9S/T\text{-}A)CreERT2}$ lines. For lineage tracing experiments, the mice were heterozygous for the reporter gene ($Rosa26^{tdTom/+}$). All animal experiments were carried out in accord with the guidelines of the UK Home Office, under the authority of a Home Office project license approved by the Animal Welfare and Ethical Review Body at the CRUK Cambridge Institute, University of Cambridge.

### Cell Lines

DLD-1 (human colon adenocarcinoma, male) cells, modified with the Flp-In T-Rex system (Thermo Fisher), were used in the study. The cell line authentication was carried out using Single Tandem Repeat (STR) genotyping. Tests were performed routinely to confirm mycoplasma-negative status of the cells. The cells were maintained in Dulbecco's Modified Eagle Medium (DMEM) with L-glutamine. Medium was supplemented with 10% Tet System-approved fetal bovine serum (FBS, Clontech). The cells were cultured under standard conditions (5% $CO_2$, 37°C).

## METHOD DETAILS

### Cloning of mouse knock-in constructs

For generation of mouse knock-ins *Atoh1* locus and homology arms were amplified from a baterial artificial chromosome (BAC) RP24-77K22 (BACPAC Resources Centre). The targeting construct was assembled by a combination of seamless cloning (In-Fusion, Clontech) and restriction digest and ligation. For this a loxP site was introduced into 5′UTR of Atoh1 via PCR amplification. A neomycin cassette was inserted such that the 3′UTR was not disrupted. The CreER$^{T2}$-hCD2-3′UTR was generated via gene synthesis service (Integrated DNA Technologies). The *Atoh1* sequence (*Atoh1*$^{(WT)}$ or *Atoh1*$^{(9S/T-A)}$) was merged with this construct, and then ligated with Atoh1 vector containing the homology arms. The targeting vector sequence was verified by Sanger sequencing and linearized by SwaI enzyme before transfecting into ES cells. The final inserted sequence is available on request.

### ES cell targeting

Electroporation of the targeting construct into mouse ES cells was conducted by the CRUK CI Transgenic Core. ES cells were positively selected with G418. Correct integration of the construct was verified by long range PCR (SequalPrep, Thermo Fisher) according to the manufacturer's instructions. Left integration arm was detected using a forward primer 5′-GGA CAG GCG GGA ACC ACA GA-3′ and a reverse primer 5′-TTG TCA ACA CGA GCT GGT CGA A-3′. Right integration arm was amplified using the following set of primers: forward 5′- CAA CAC AAC CCT GAC CTG TG-3′, and reverse 5′-CCC TAA CCA GTG TGC CCT TA-3′. Copy number of the clones was determined by qPCR of the neomycin selection cassette via a commercial genotyping service provider (Transnetyx). Single copy ES cell clones were taken forward for blastocyst injection, and chimeric mice were generated. Following successful germline transmission, the mice heterozygous for the targeting construct were crossed onto *PGK-Cre* line (Lallemand et al., 1998) in order to remove both the neo selection cassette and the endogenous *Atoh1* locus at the same time. A constitutively active *Atoh1-P2A-CreER*$^{T2}$ allele was generated in this process.

### Mouse genotyping

Genotyping was carried out by Transnetyx. Manual genotyping by PCR was used to distinguish between homozygous and heterozygous Atoh1 animals. The following primers were used: forward 5′-TTT GTT GTT GTT GTT CGG GG-3′; reverse 5′-TCT TTT ACC TCA GCC CAC TCT T-3′.

### Creation of doxycycline inducible DLD-1 cells

To generate an inducible stable cell line, a DLD-1 Flp-In T-Rex cell line containing a single Frt site was obtained (a generous gift from Prof Stephen Taylor, University of Manchester). Atoh1 construct in a pcDNA 5/FRT/TO vector (Thermo Fisher) was co-transfected with pOG44 (Flp recombinase-expressing plasmid) in a 1:9 ratio (JetPrime, Polyplus transfection). Cells were washed 24 h after transfection, and fresh medium was added. Two days after transfection, the cells were split at a low confluence (less than 25%), and hygromycin (400 μg/mL) was added to the trypsinised cells. Fresh medium was added to the cells every 3-4 days, until the non-transfected cells died off, and foci of surviving cells could be visualized. Doxycycline (100 ng/mL, Sigma) was added to the culture 24 h after seeding, to induce expression of the gene of interest.

Validation of the correct recombination of the construct was carried out by PCR. Left integration arm was detected by using the following set of primers: forward 5′-AGT CAG CAA CCA TAG TCC CG-3′; reverse 5′- TTC TGC GGG CGA TTT GTG TA-3′. Correct integration on the 3′ end of the construct was done using a forward primer 5′-TAA ACG GCC ACA AGT TCA GC-3′, and a reverse primer 5′-CGG GCC TCT TCG CTA TTA CG-3′. Parental DLD-1 Flp-In T-Rex cell line was used as a negative control.

The expected loss of β-galactosidase activity on targeting was verified by X-Gal (5-bromo-4-chloro-3-indolyl-β-D-galactopyranoside) staining of fixed cells. To validate that the constructs were integrated as a single copy in the genome, copy number qPCR was employed. Copy number TaqMan probes detected HygR (Mr00661678_cn) and used a reference copy number assay for RNase P detection.

### Treatment of animals

Induction of CreER$^{T2}$ in animals was carried out using the free base tamoxifen (Sigma) dissolved in ethanol/oil (1:9). The animals received 3 mg tamoxifen via an intra-peritoneal injection in all experiments. To define Atoh1 secretory signature, the mice were injected with 1 mg tamoxifen per day on 3 consecutive days for maximal labeling of all secretory lineages.

SI injury was induced by exposing animals to whole-body irradiation (6 Gy). To induce colon-specific injury, mice were given 1.5% DSS (MP Biomedicals) in drinking water for 5 days. DSS was replaced every two days during the treatment. To induce lineage tracing and ablate Lgr5$^{+}$ cells in Lgr5$^{DTR}$ mice, the animals first received 3 mg tamoxifen i.p., followed by an i.p. injection of DT in saline (50 μg/kg) 6 h later.

### Crypt fractionation and single cell preparation

SI (proximal 15 cm) and colon were dissected, flushed with PBS, everted and fed onto a glass rod spiral. They were incubated at 37°C in Hank's Balanced Salt Solution (HBSS) without Ca$^{+2}$ and Mg$^{+2}$, containing 10 μM EDTA and 10 mM NaOH. Crypt release was facilitated using a vibrating stirrer (Chemap). Samples were incubated for 1 h and pulsed every 10 min. Fractions were collected after each pulse, and fresh solution added. Crypt-enriched fractions were pooled and washed in cold 2% FBS/PBS. Fraction 1 (villus-enriched)

was discarded. Pooled fractions were resuspended in 0.05% trypsin and incubated for 7 min at 37°C, shaking every 1 min. Single cells were then filtered through a 70 μm mesh, and washed twice in 2% FBS/PBS.

### Flow cytometry

Single cell suspension obtained by trypsin treatment was washed and incubated with an anti-mouse CD326 (EpCAM) AlexaFluor 647 antibody (1:2,000, clone G8.8, Biolegend). DAPI (10 μg/mL) was added to distinguish between live and dead cells. Flow sorting was carried out on a BD FACS Aria SORP (BD Biosciences), using appropriate single-stained and unstained controls.

### Whole-mount preparation

Tissue was cut open, pinned out luminal side up, and fixed for 3 h at room temperature in ice-cold 4% PFA in PBS (pH 7.4). Whole-mounts were washed with PBS, and incubated with demucifying solution (3 mg/mL dithiothreitol (DTT), 20% ethanol, 10% glycerol, 0.6% NaCl, 10 mM Tris, pH 8.2) for 20 min, and mucus removed by washing with PBS.

### Whole-mount scanning and quantification

The tdTom fluorescence in colon whole-mounts was detected using Amersham Typhoon 5 laser scanner (GE Healthcare) at a 10 μm resolution. The tdTom$^+$ foci were scored manually in Fiji. Mid and distal colon were scored only as the shape of the proximal colon prevented confident assessment of tdTom$^+$ patches.

### Antibody staining

For staining whole-mount sections of 2 cm in length were excised, washed in 0.1% PBS-T for 2 days, and blocked in 10% donkey serum in PBS overnight at 4°C, protected from light. Samples were then incubated with an anti-mouse CD326 (EpCAM) AlexaFluor 647 antibody (1:100, clone G8.8, Biolegend) in 10% donkey serum in PBS for 3 days. Finally, the tissue was washed with PBS-T for 1 day.

### Quantification of crypts in whole-mounts

Imaging was done on a TCS SP5 confocal microscope (Leica). Images were analyzed using Fiji. For SI, a minimum of 2,500 crypts per animal was scored. For colon, at least 900 crypts per mouse were scored. For the low-power analysis of clonal events, tdTom$^+$ clones were scored across the entire length of the SI whole-mounts using a stereomicroscope (Nikon).

### Immunostainings

For immunohistochemistry SI and colon were opened and fixed for 24 h in 10% neutral buffered formaldehyde in PBS. The tissue was paraffin embedded and sectioned by the CRUK CI Histopathology core. Haematoxylin and eosin staining was performed using an automated ST5020 Multistainer (Leica Biosystems). Alcian Blue and Periodic Acid/Schiff staining was carried out by the CI Histopathology Core. Briefly, slides were incubated in Alcian Blue for 10 min, and washed in water. They were then incubated in 0.5% periodic acid for 5 min, and washed 3 times. Slides were incubated in Schiff's reagent for 15 min, washed 3 times, and counterstained with Mayer's Haematoxylin.

BrdU and lysozyme immunohistochemistry was carried out using a Bond Max autostainer (Leica), with a proteinase K antigen retrieval. Slides were blocked with 3% hydrogen peroxide, followed by incubation in Avidin/Biotin Blocking Kit (Vector Laboratories). BrdU was detected using a sheep anti-BrdU antibody (1:500, Abcam ab1893). Rabbit anti-lysozyme antibody (1:500, Dako A0099) was used for lysozyme staining. Secondary antibodies in the two cases were biotinylated donkey anti-sheep (1:250, Jackson ImmunoResearch 713-066-147) and biotinylated donkey anti-rabbit (1:250, Jackson ImmunoResearch 711-065-152), respectively. Slides were incubated with Streptavidin coupled with horseradish peroxidase (HRP), and color developed using diaminobenzidine (DAB) and DAB Enhancer (Leica).

Synaptophysin and Chromogranin A detection was carried out by manual IHC. Antigen retrieval was performed with 10 mM citrate buffer (pH 6.0) in a pressurised heating chamber. Tissue sections were incubated with rabbit anti-Chromogranin A antibody (1:500, Abcam ab15160), rabbit anti-Synaptophysin antibody (1:300, Millipore AB9272), overnight at 4°C. Slides were incubated with biotinylated donkey anti-rabbit secondary antibody (1:500, Jackson ImmunoResearch 711-065-152). Streptavidin-HRP conjugate (Vector Laboratories) was added onto the slides and incubated for 30 min. DAB Chromogen substrate (Dako) was added for dye development. Counterstaining and dehydration was performed on the ST5020 Multistainer (Leica) followed by coverslipping.

For immunofluorescence tissue was excised and fixed for 48 h in 4% PFA in PBS at 4°C, after which it was transferred to 20% sucrose solution. After cryosectioning antigen retrieval where needed was accomplished by incubating the slides in 1% SDS for 5 min. Blocking was performed with 5% donkey serum. Following a wash primary antibodies were added and incubated overnight at 4°C. The following primary antibodies were used: rabbit FITC-anti-Lyz (1:400, Dako, F037201), rabbit anti-Muc2 (1:50, Santa Cruz, sc-15334), rabbit anti-ChgA (1:100, Abcam, ab15160), and rabbit anti-Dclk1 antibody (1:1000, Abcam, ab31704). Secondary detection was with AlexaFluor 488 donkey anti-rabbit secondary antibody (1:500, Thermo Fisher, A-21206). Alkaline phosphatase activity was detected using Blue AP kit (Vector Laboratories). Sections were covered with Prolong Gold with DAPI (Life Technologies). Fluorescent imaging was carried out on a TCS SP5 confocal microscope (Leica).

CellPress

## Single molecule FISH

Harvested SI and colon tissues were flushed with cold 4% formaldehyde (FA) in PBS and incubated first in 4% FA/PBS for 3 hours, then in 30% sucrose in 4% FA/PBS overnight at 4°C with constant agitation. Fixed tissues were embedded in OCT. Quantification of co-expression was achieved by smFISH. Probe library design, hybridization procedures, and imaging settings were carried out according to published methods (Itzkovitz et al., 2011; Lyubimova et al., 2013). A Nikon-Ti-E inverted fluorescence microscope equipped with a Photometrics Pixis 1024 CCD camera was used to image a 10 μm cryo-section. A stack of 30 frames with 0.3 μm intervals was acquired to allow 3D cell imaging. FITC-conjugated antibody for E-cadherin was added to the hybridization mix and used to visualize cell borders. Detection of cells that were positive for Lgr5 transcripts, Atoh1 transcripts or both was performed manually with Fiji.

## Analysis of gut sections

Stained longitudinal sections of the SI and colon were visualized and positive cells scored manually. $BrdU^+$ and negative nuclei were scored in complete half-crypt sections. $Lysozyme^+$ cells were counted per whole crypt section. Alcian Blue and $PAS^+$ cells were counted in complete half-villus sections, between the crypt neck and the tip of the villus. Cells in which the stain was clearly associated with a corresponding nucleus were marked as positive. Chromogranin $A^+$ and $synaptophysin^+$ cells were scored per complete half-crypt-villus section. Positive and negative crypts were scored, and results expressed as a frequency of positive cells.

## Colon ulceration scoring

H&E-stained sections of colons were scanned on Aperio slide scanner (Leica Biosystems), and analyzed using eSlide Manager (Leica Biosystems). Ulceration was defined as a region of a complete loss of crypt architecture and high cellularity.

## RNA isolation

For gene expression analysis by qPCR, cells were lysed and RNA isolated using RNeasy Mini Plus kit (QIAGEN). For sequencing, total RNA was isolated from flow-sorted cells using RNeasy Micro Plus kit (QIAGEN).

## Gene expression analysis

RNA was converted into cDNA (iScript cDNA synthesis kit, BioRad), and gene expression was analyzed using TaqMan gene expression probes (Thermo Fisher). The following probes were used: Atoh1 (Mm00476035_s1), Muc2 (Hs00894053_g1), Tff3 (Hs00902278_m1), Spdef (Hs01026050_m1), Dll4 (Hs00184092_m1), Rassf4 (Hs00604698_m1), Gapdh (Hs02758991_g1). All TaqMan assays are listed in Table S3.

## RNA sequencing

Samples for RNA sequencing were collected 24 h post-tamoxifen induction (3 mg i.p. injection). The tissue was fractionated as described above and cells prepared for flow cytometry. The cells were stained and sorted in the same way as for other experiments, as noted above. $EpCAM^+tdTom^+$ live cells were collected directly into the lysis buffer and RNA was extracted immediately following the sort (RNeasy Micro Plus Kit, QIAGEN). RNA quality was assessed on a 2100 Bioanalyser instrument (Agilent), according to the manufacturer's instructions. The libraries were prepared using TruSeq Stranded mRNA Library Prep Kit (Illumina) and sequenced as 50 bp single-end reads on the Illumina HiSeq 4000 system.

## Western blotting

Protein extracts for SDS-PAGE were prepared by lysing the cells with RIPA buffer containing protease and phosphatase inhibitor cocktail (Thermo Fisher). Mouse anti-ATOH1 antibody (1:100, Developmental Studies Hybridoma Bank) and a rabbit anti-β-A antibody (1:5,000, ab8227, Abcam) were used. Fluorescent secondary antibodies were used (Li-Cor, goat anti-mouse 800LT (1:5,000), goat anti-rabbit 680LT (1:20,000)). For some experiments, protein extracts were incubated with λ phosphatase (New England Biolabs) prior to western blotting, according to the manufacturer's instructions.

## *In vitro* kinase assay

The assay was performed as previously described (Azzarelli et al., 2017), with minor modifications. HA-tagged WT and mutant ATOH1 were *in vitro* translated (TNT® Quick Coupled Transcription/Translation Systems, Promega) in the presence of LiCl (800 mM) to reduce potential phosphorylation in reticulocyte lysate. Samples were incubated with human recombinant CDK/Cyclins (0.25 μM final concentration) in the presence of 10 μM ATP for 1 h at 30°C. Proteins were separated on Phos-tag gels (Alpha Laboratories, 7.5% acrylamide, 50 μM phos-tag PAGE, Wako) and immunoblotted with rat anti-HA-Peroxidase (1:5000, Roche).

## Cell proliferation and cell cycle analysis

Cell proliferation was assessed by an automated live-cell imaging system (IncuCyte ZOOM, Essen Bioscience). For cell cycle analysis, the cells were trypsinised, washed, fixed with ethanol, and stained with propidium iodide prior to flow cytometry.

## RNA sequencing analysis

The reads were aligned to the mouse reference genome [GRCm38] using TopHat2 aligner (Kim et al., 2013). Differentially expressed gene lists were generated using DESeq2 package from Bioconductor (Love et al., 2014).

## Secretory signature gene list

The list of differentially expressed genes ($p < 0.01$) was generated by comparing the transcripts from $tdTom^{+}$ and $tdTom^{-}$ cells of *$Atoh1^{(WT)CreERT2}$ $Rosa26^{tdTom/+}$* mice following tamoxifen. Upregulated genes in $tdTom^{+}$ cells were selected to define a secretory signature in the small intestine and colon (Table S1). The top 500 upregulated, differentially expressed genes were used to perform the Gene Set Enrichment Analysis (GSEA).

## Gene Set Enrichment Analysis (GSEA)

This analysis was performed using the GSEA software from the Broad Institute (http://software.broadinstitute.org/gsea/index.jsp) (Subramanian et al., 2005). The list comprised all differentially expressed and non-differentially expressed genes from the *9S/T-A* v *WT* comparison in SI and colon, respectively. This gene list was probed with the previously generated secretory signatures (top 500 upregulated genes), and the published $Atoh1^{+}$ gene signatures for ileum, colon (Lo et al., 2016), and intestinal stem cells (Muñoz et al., 2012).

# QUANTIFICATION AND STATISTICAL ANALYSIS

## Computational analysis

The process by which crypt stem cells replace each occurs in a random though predictable manner. This behavior can be modeled via a stochastic birth-death process (Lopez-Garcia et al., 2010; Snippert et al., 2010). The model was derived to model experiments where a single stem cell is labeled in a handful of crypts. As the number of initially labeled crypts was not of interest and to bypass any variability coming from the initial induction, make the different time points comparable, the equations were rescaled to account for only the surviving clones. Here we know the parameters of the stem cell dynamics (Kozar et al., 2013; Vermeulen et al., 2013), and would like to know the starting number of labeled stem cells per crypt and the number of labeled crypts.

For this analysis we use the equations described previously (Lopez-Garcia et al., 2010; Snippert et al., 2010), reproduced below. The probability of a crypt having clone of size n (for $0 < n < N$) at time t is:

$$p_n(t)\frac{2}{N}\sum_{m=1}^{N-1}\sin\left(\frac{\pi m}{N}\right)\sin\left(\frac{\pi mn}{N}\right)e^{-4\lambda\,\sin^2\left(\frac{\pi m}{2N}\right)t} \quad \text{(Equation 1)}$$

Here n is the number of labeled stem cells, N is the total number of stem cells, λ is the rate of stem cell replacement. And for the probability of all stem cells labeled we have:

$$p_N(t)\frac{2}{N}\sum_{m=1}^{N-1}(-1)^{m=+1}\cos^2\left(\frac{\pi m}{2N}\right)\left(1-e^{-4\lambda\,\sin^2\left(\frac{\pi m}{2N}\right)t}\right) \quad \text{(Equation 2)}$$

These equations assume the initial conditions of one labeled stem cell at $t = 0$. The starting labeled stem cells were chosen randomly at the beginning of each simulation.

The values we observe for the clonal frequencies are substantially lower than what the model would predict, suggesting that not all crypts have labeled stem cells. In order to find out the fraction of labeled crypts $v$ we use a mixture model:

$$Q_n(t) = (1-v)\delta_{0,n} + vP_n(t) \quad \text{(Equation 3)}$$

Where $Q_n(t)$ is the probability that a randomly selected crypt has a clone of size n labeled stem cells at time t. We use the values of N, λ and $\tau$ from Kozar et al. (2013) and Vermeulen et al. (2013) and estimate $v$.

## Model fitting

For every mouse, at day 30 we count the number of clones ($k_i$) and the number of crypts ($C_i$). We use a hierarchical model to capture the mouse to mouse variability. The statistical model is a follows

$$\kappa_i \sim Binomial(C_i, R_i(30-\tau)) \quad \text{(Equation 4)}$$

$$R_i(30-\tau) \sim Student\_t(\eta, Q(30-\tau), \sigma) \quad \text{(Equation 5)}$$

Here $R_i$ is truncated to [0, 1]. For the SI no distinction is made in clone size, so Q is the sum of all $Q_n$ and for the colon we use only the full clones for fitting $Q = Q_{N.}$

The priors on the population parameters are:

$$\eta \sim Gamma(2.0.1) \quad \text{(Equation 6)}$$

$$\sigma \sim Gamma(0.01, 0.01) \quad \text{(Equation 7)}$$

The prior on the mixing coefficient is

$$\eta \sim Beta(1/2, 1/2) \quad \text{(Equation 8)}$$

The posterior was derived via MCMC using Rstan (Carpenter et al., 2017). For the proximal and distal SI we used is $\tau = 5$ as the clones were measured in ribbons coming out of the crypt, which take a few days to emerge from the crypt base. Whereas for the colon we used $\tau = 1$. The parameters used were $\tau = 0.1$, N = 5 for proximal SI, $\tau = 0.2$, N = 6 for distal and $\tau = 0.3$, N = 7 for colon.

### Statistical analysis

Statistical tests were not used to predetermine sample size. Randomization was not performed to allocate samples/animals to experimental groups. Blinding was performed for quantifications in Figures 3F and 3G, as well as Figures S2B–S2F. Data analysis was performed using GraphPad Prism software or R package.

## DATA AND SOFTWARE AVAILABILITY

The accession number for the RNA sequencing data reported in this paper is GEO: GSE115416. Mendeley Dataset of original data can be accessed at https://doi.org/10.17632/vgvdv5b949.1.

Cell Stem Cell, Volume *23*

## Supplemental Information

# Phospho-regulation of ATOH1 Is Required for Plasticity of Secretory Progenitors and Tissue Regeneration

**Goran Tomic, Edward Morrissey, Sarah Kozar, Shani Ben-Moshe, Alice Hoyle, Roberta Azzarelli, Richard Kemp, Chandra Sekhar Reddy Chilamakuri, Shalev Itzkovitz, Anna Philpott, and Douglas J. Winton**

# Figure S1. Related to Figure 1.

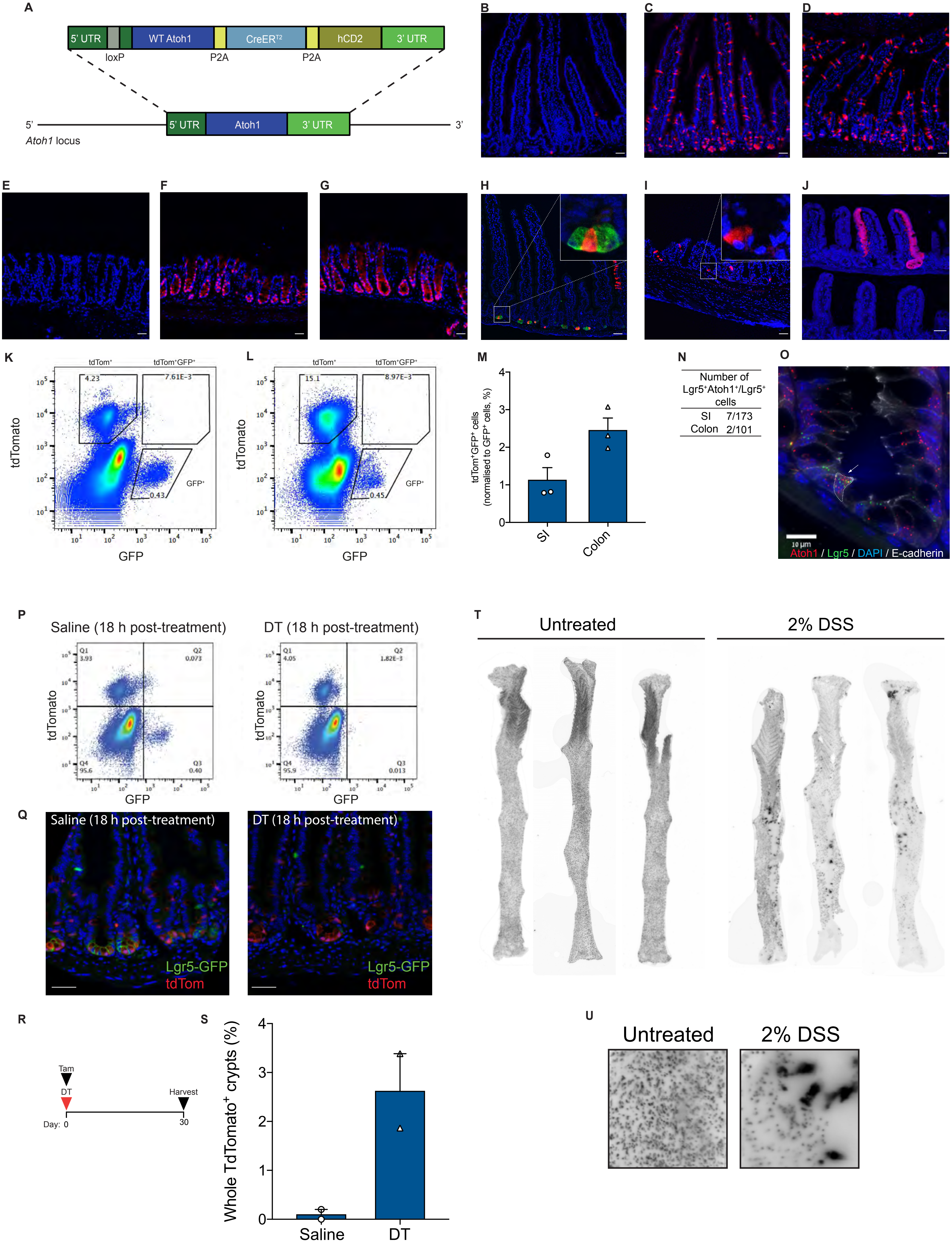

**Figure S1. Generation of *Atoh1$^{(WT)CreERT2}$* mouse model and validation of lineage tracing in *R26R$^{tdTom}$* reporter line in homeostasis and injury, Related to Figure 1**

(A) Schematic of the lineage tracing construct inserted into *Atoh1* genomic locus. (B, E) SI (B) and colon (E) of the uninduced animals. (C, F) Reporter positive cells in the SI (C) and colon (F), 24 h post-tamoxifen. (D, G) $tdTom^+$ cells in the SI (D) and colon (G), 4 days post-tamoxifen. (H) Long-lived $Lyz^+$ Paneth cells observed 30 days post-induction. (I) Long-lived reporter positive cells in the upper colon detected 30 days post-tamoxifen. (J) Labelled clonal event in the SI, 4 months post-tamoxifen. (K, L) Representative FACS graphs of $tdTom^+GFP^+$ cells in SI (K), and colon (L), 24 hours post-tamoxifen. (M) Quantification of $tdTom^+GFP^+$ cells in SI and colon. (N, O). Quantification (N) and a representative image (O) of Atoh1/Lgr5 transcript-positive cells in *WT* SI and colon (n=3). (P, Q) Ablation of Lgr5-$GFP^+$ cells following administration of DT analysed by FACS (P) and microscopy (Q). (R) Schematic of the induction/injury protocol. (S) Quantification of $tdTom^+$ clones in the SI without or with DT treatment (n=2 for each group). (T, U) Images of scanned colons showing $tdTom^+$ crypts and multicrypt patches (MCPs) used for scoring in Figure 1U. Scale bars, 50 μm (B-J, Q); 10 μm (O). DT, diphtheria toxin.

Figure S2. Related to Figure 2.

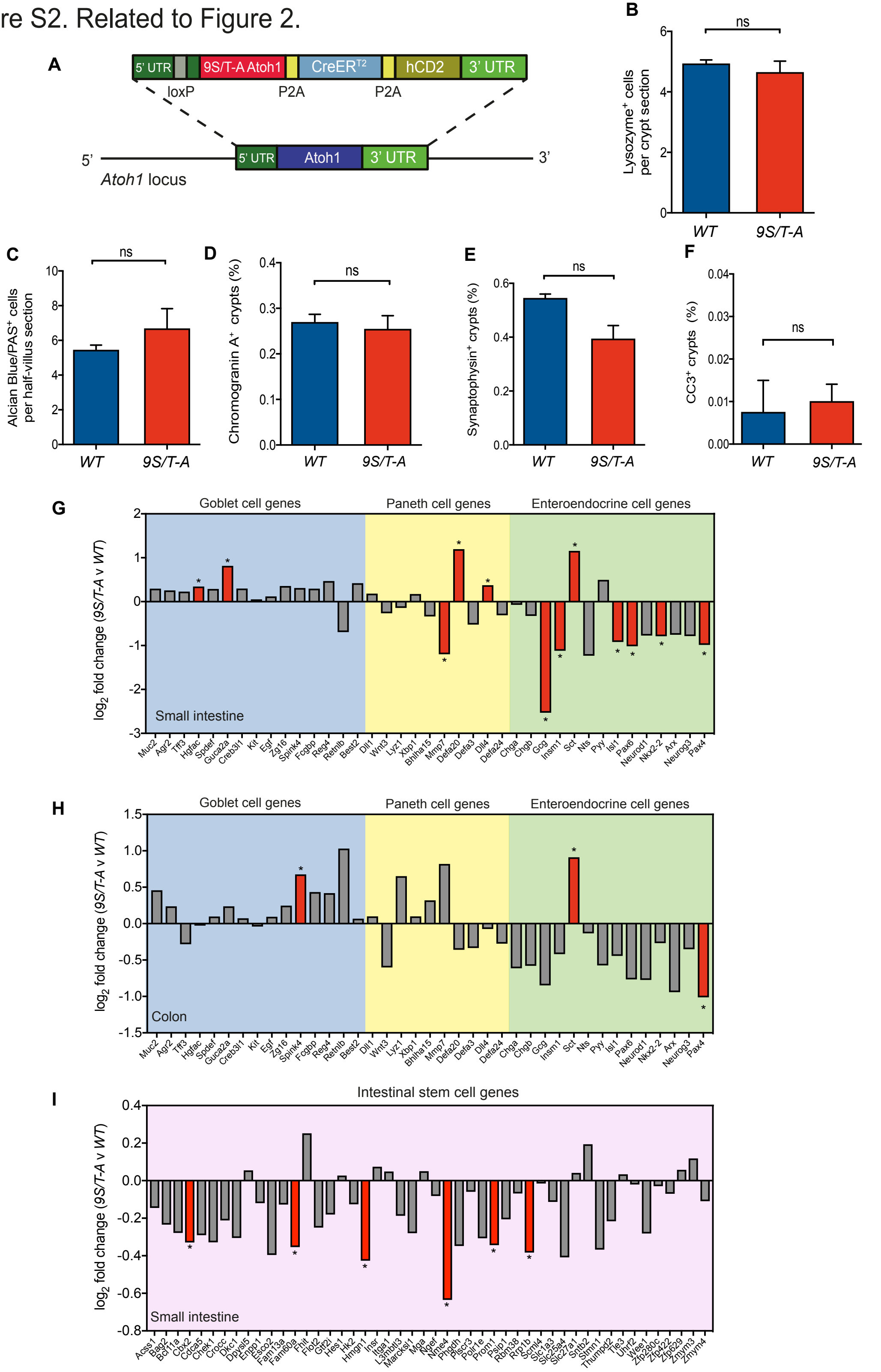

**Figure S2. *Atoh1$^{(9S/T\text{-}A)CreERT2}$* mice do not exhibit abnormal intestinal secretory cell numbers, but show a change in gene expression profiles, Related to Figure 2**

(A) Schematic representation of the *Atoh1$^{(9S/T\text{-}A)CreERT2}$* inserted into the *Atoh1* locus. (B-F) Quantification of differentiated small intestinal secretory lineage cells and apoptotic cells shows no difference between the genotypes. Paneth cells (B), goblet cells (C), enteroendocrine cells (D, E), and apoptotic cells (F) were scored (n=4 mice per group). (G-I) Expression of secretory genes selected based on known lineage-restricted pattern (Lo et al., 2017) in SI (G) and colon (H) of 9S/T-A Atoh1-expressing cells. (I) Expression of intestinal stem cell genes (Muñoz et al., 2012) in SI of 9S/T-A Atoh1-expressing cells. Genes that are significantly differentially expressed (FDR < 0.1) are shown in red, labelled with an asterisk. The results were generated from n=6 for both *WT* and *9S/T-A* groups.

Figure S3. Related to Figure 3.

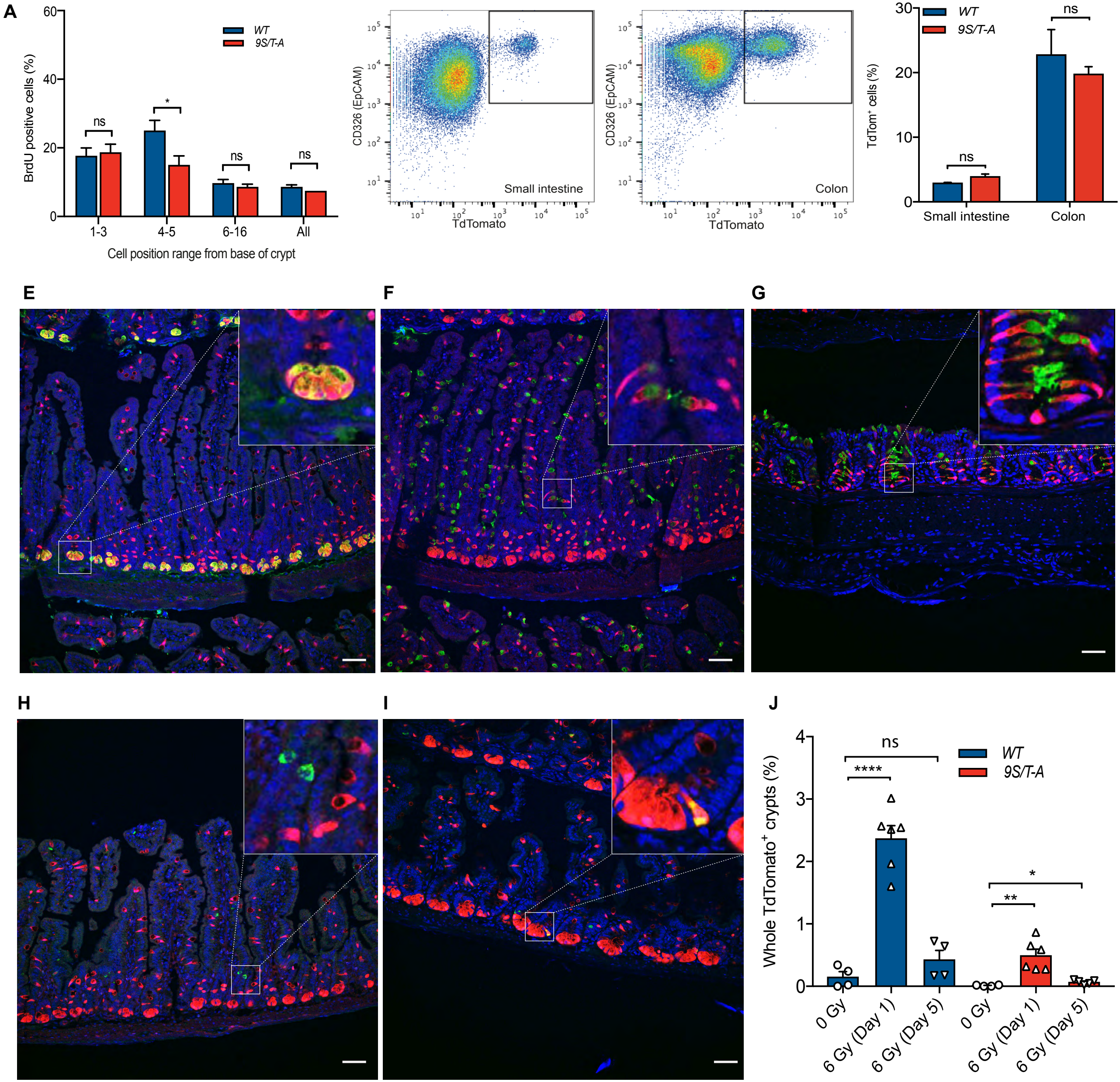

**Figure S3.** ***Atoh1$^{(9S/T\text{-}A)CreERT2}$*** **mice are phenotypically normal, but have a reduced clonogenic potential, Related to Figure 3.** (A) BrdU labelling index for a range of cell positions in colonic crypts (n=100 crypts, 4 mice per genotype; mean±s.e.m; * P=0.0101). (B, C) Representative flow cytometry plots of reporter positive cells in the SI (B) and colon (C). (D) Quantification of tdTom$^+$ cells in the SI and colon of the two mouse lines (n=3 per group, Mann Whitney test). (E-I) tdTom-labelling pattern in *9S/T-A* is identical to that of *WT*. Lyz$^+$ Paneth cells (E), Muc2$^+$ goblet cells in the SI (F), and colon (G) are positive for the reporter 24 h post-tamoxifen. ChgA$^+$ enteroendocrine cells do not co-label with tdTom after 24 h (H), but acquire the reporter by day 4 post-induction (I). (J) Comparison of the response to irradiation in WT Atoh1 and 9S/T-A small intestine. Scale bars, 50 µm. The data for WT Atoh1 are repeated from Figure 1O (Welch's T-test, **** P<0.001, ** P=0.0045, * P= 0.0122).

Figure S4. Related to Figure 4.

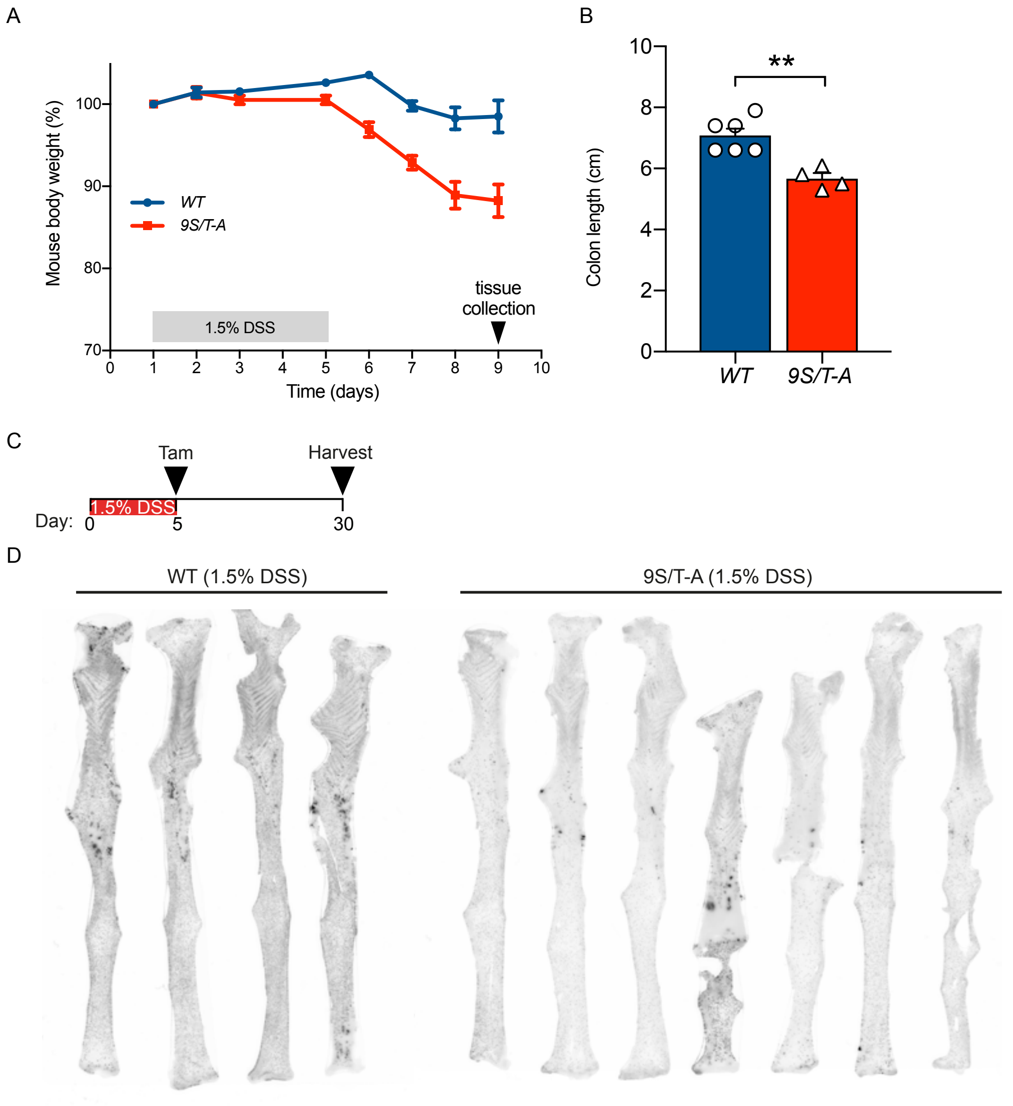


**Figure S4. Characterisation of chemical colitis in *Atoh1*$^{(9S/T-A)CreERT2}$ mice, Related to Figure 4**

(A) Mouse body weight difference between *WT* and *9S/T-A* mice on day 9 (n=6 (*WT*), n=4 (*9S/T-A*); related to Figure 4B and 4C). (B) Colon length following DSS treatment (n=6 (*WT*), n=4 (*9S/T-A*), mean±s.e.m, ** P=0.0048). (C) Lineage tracing protocol following DSS colitis. (D) Images of scanned colons used for quantification in Figures 4G and 4H.
